# Minimal Out-of-Equilibrium Metabolism for Synthetic Cells: A Membrane Perspective

**DOI:** 10.1021/acssynbio.3c00062

**Published:** 2023-04-07

**Authors:** Eleonora Bailoni, Michele Partipilo, Jelmer Coenradij, Douwe A. J. Grundel, Dirk J. Slotboom, Bert Poolman

**Affiliations:** ^†^Department of Biochemistry and ^‡^Molecular Systems Biology, Groningen Biomolecular Sciences and Biotechnology Institute, University of Groningen, Nijenborgh 4, 9747 AG Groningen, The Netherlands

**Keywords:** bottom-up synthetic cells, minimal metabolism, JCVI-syn3a, out-of-equilibrium, energy conservation, metabolite transport, membrane composition, physicochemical homeostasis

## Abstract

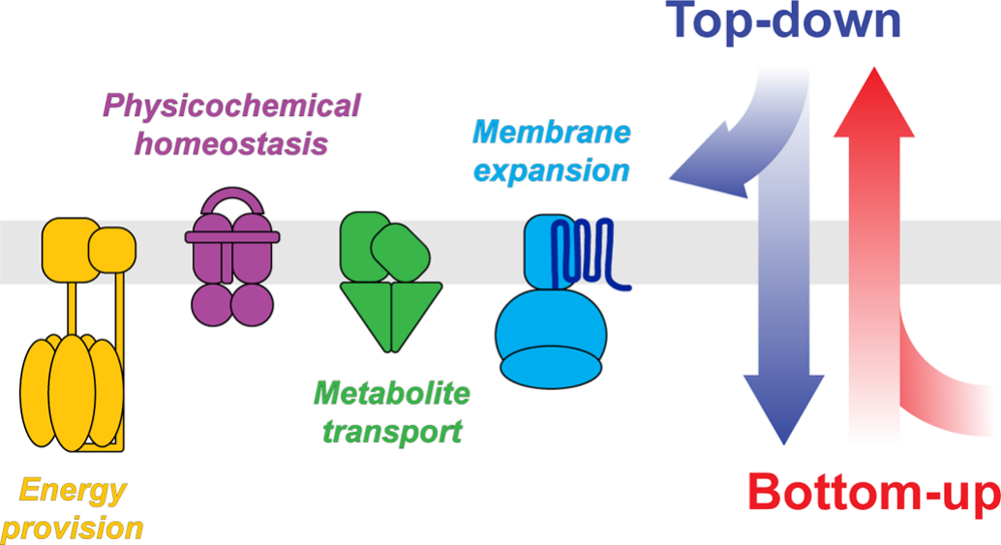

Life-like systems need to maintain a basal metabolism, which includes importing a variety of building blocks required for macromolecule synthesis, exporting dead-end products, and recycling cofactors and metabolic intermediates, while maintaining steady internal physical and chemical conditions (physicochemical homeostasis). A compartment, such as a unilamellar vesicle, functionalized with membrane-embedded transport proteins and metabolic enzymes encapsulated in the lumen meets these requirements. Here, we identify four modules designed for a minimal metabolism in a synthetic cell with a lipid bilayer boundary: energy provision and conversion, physicochemical homeostasis, metabolite transport, and membrane expansion. We review design strategies that can be used to fulfill these functions with a focus on the lipid and membrane protein composition of a cell. We compare our bottom-up design with the equivalent essential modules of JCVI-syn3a, a top-down genome-minimized living cell with a size comparable to that of large unilamellar vesicles. Finally, we discuss the bottlenecks related to the insertion of a complex mixture of membrane proteins into lipid bilayers and provide a semiquantitative estimate of the relative surface area and lipid-to-protein mass ratios (i.e., the minimal number of membrane proteins) that are required for the construction of a synthetic cell.

## 1 Introduction

Life exists away from thermodynamic equilibrium. In fact, the properties and behavior of cellular systems are largely governed by the kinetics of fuel and building block supply rather than by their thermodynamic stability. In living organisms, the out-of-equilibrium state is maintained within a confined space bounded by a semipermeable membrane.^1,2^ Besides defining the cell content, the membrane also establishes and exploits (electro)chemical gradients via embedded integral membrane proteins, i.e., energy-transducing machineries including, e.g., ion channels and solute transporters. Their concerted action ensures an out-of-equilibrium state by importing fuel molecules or building blocks for biosynthetic purposes and by exporting waste products that would otherwise become harmful in the lumen.

Within the cellular boundary, a set of catalyzed chemical reactions collectively termed metabolism (that is, biosynthesis, energy conservation, central carbon metabolism, membrane transport, etc.) enables cells to remain out of equilibrium. It is therefore not surprising that a large portion of the gene products is dedicated to sustaining metabolic activity.^3^ In bacteria, the fraction of metabolism-related genes ranges from 35% in *Mycoplasma pneumoniae*, a pathogen with limited metabolic functions, up to 47% in the model organism *Escherichia coli*. JCVI-syn3a, the (known) living organism with the simplest genetic makeup, also employs one-third of its genes for metabolism and physicochemical homeostasis.^3^

**Figure 1:**
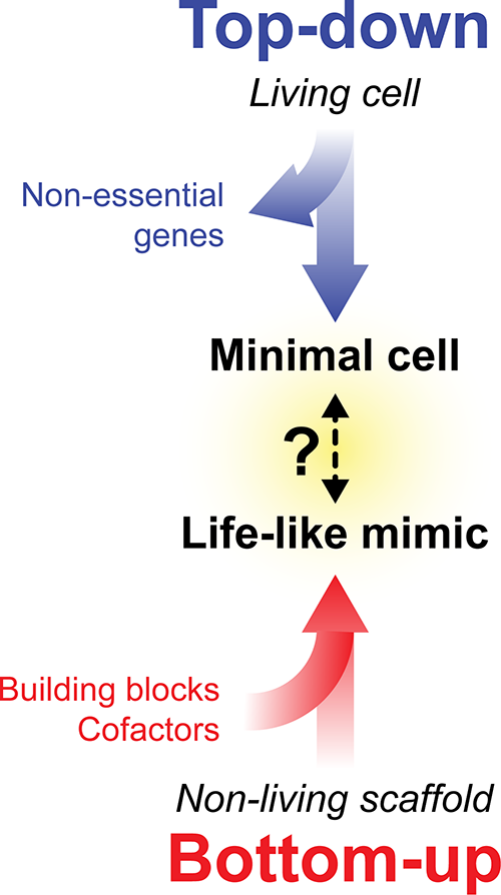
**Engineering of synthetic cells.** Top-down approaches create minimal cells by deleting nonessential sequences from the genomes of living organisms. These are then transplanted into host cells devoid of genetic material. Bottom-up strategies assemble nonliving building blocks into synthetic systems to obtain life-like properties.

JCVI-syn3a was developed by the sequential knockout of nonessential genes from *Mycoplasma mycoides capri*.^3^ This top-down approach, also applied to other model organisms such as *Bacillus subtilis*(4) and *Escherichia coli*,^5^ aims to identify a minimal set of essential genes, in order to understand life at the molecular level. Approximately one-third of the essential and quasi-essential gene products of JCVI-syn3a do not have a known or predictable function,^6^ which necessitates the use of complementary approaches to fully understand the minimal requirements of life-like systems. The bottom-up assembly of a minimal synthetic cell from well-characterized molecular building blocks would lead to a highly defined and controllable life-like system and would provide such complementary insight, but the path toward a fully functioning cell is long (Figure 1).

The engineering of minimal synthetic cells stripped from nonessential functions is currently an active area of research with many scientific and technological challenges.^7−10^ Depending on the research field or application, conceptually different minimal or synthetic cells have been designed. For example, the study of plausible scenarios for the onset of life on Earth^11,12^ requires that the building blocks are compatible with prebiotic environmental conditions. Alternatively, synthetic cells are exploited as a platform to study the universal principles that govern life at the molecular level,^13,14^ these systems being simpler than existing living organisms. For this purpose, components (e.g., DNA, proteins, lipids, etc.) can be sourced (and engineered) from different organisms, in order to obtain the desired functions. Artificial parts designed de novo in the laboratory are also used to reproduce specific aspects of living organisms.^15,16^ However, fully autonomous synthetic cells ultimately rely on templates encoding the instructions for their self-reproduction, growth, and division, which are executed by the synthetic cell machinery. While nonbiological components and approaches (nonenzymatic reactions) are very important, no artificial alternative has to date been created to replicate such components and replace the biological system based on nucleic acids. Therefore, we argue that the construction of autonomous synthetic systems aimed to recapitulate the fundamental aspects of living cells will rely on components that can be genetically encoded.

We envision future synthetic cells as minimal self-sustained molecular assemblies that operate as selectively open systems^17^ (Box 1). They grow and ultimately divide into two new entities that possess the same essential properties of physicochemical homeostasis and self-replication. Mechanisms of evolvability could also be implemented to ensure adaptability to different environmental conditions by the acquisition of advantageous phenotype(s) in the new cells.^18,19^ Importantly, the design of synthetic cells can draw inspiration from the minimized genome of JCVI-syn3a, which could be regarded as the best benchmark organism for bottom-up efforts. In fact, a mixed top-down/bottom-up strategy is a powerful approach toward unraveling the emergent properties of life, as cell mimics could be used to elucidate the unknown functions of JCVI-syn3a genes under controlled conditions.

Box 1 **Bottom up-synthetic cells: closed versus open systems**

**Closed systems.** Lipid vesicles allow for the transmembrane diffusion of small neutral and hydrophobic molecules, while they are highly impermeable to hydrophilic molecules. Hence, conventional lipid vesicles are essentially closed systems, and building blocks need to be encapsulated from the beginning. These systems offer very limited control of the internal reaction networks.^28^ These systems are bound to reach thermodynamic equilibrium either due to precursor depletion or byproduct accumulation.

**Open, unselective systems.** Pore-forming toxins such as cytolysin A (ClyA) from *Salmonella enterica*(29) and α-hemolysin (αHL) from *Staphylococcus aureus*(30) self-assemble into oligomeric α- and β-barrel pores, respectively. These toxins also self-insert into membrane bilayers in vitro, thereby circumventing the need for a mechanism to insert a protein into the membrane (Box 3). Next to the pore-forming toxins, a variety of nanopores has been engineered,^31^ using different polymers (e.g., DNA^15,32−35^) and finding applications in the construction of synthetic cells^36−38^ but also DNA^39^ and protein^40^ sequencing.^41^ Nonselective pores allow molecules to diffuse in or out the synthetic cell according to their concentration gradients. Toxins with pore diameters of 1.4–4.0 nm are availabe^29,30^ so that macromolecules (DNA, RNA, proteins) are retained while metabolites can enter or leave the compartment. A main disadvantage is that small molecules cannot be accumulated against their concentration gradient, which is an essential feature of living systems.

**Open, selective systems**. Reconstituting membrane transporters in lipid vesicles generates selectively open systems that can maintain an out-of-equilibrium state by accumulating specific nutrients and excreting unwanted end products. Such systems are typically driven by ATP or electrochemical ion gradients. They allow cells to grow under environmentally changing or low-nutrient conditions, and they should ultimately be reproduced in synthetic systems.

**Beyond open systems: open environment.** A selectively open system that is placed in a closed environment (e.g., a test tube) will ultimately reach equilibration, i.e., when the substrates run out and the waste products accumulate. This can be avoided by opening the external environment, that is, by introducing a continuous nutrient flow that simultaneously removes products, whereas the synthetic systems are retained (e.g., continuous flow dialysis,^42^ microfluidic traps,^43^ etc.).

**Figure 2:**
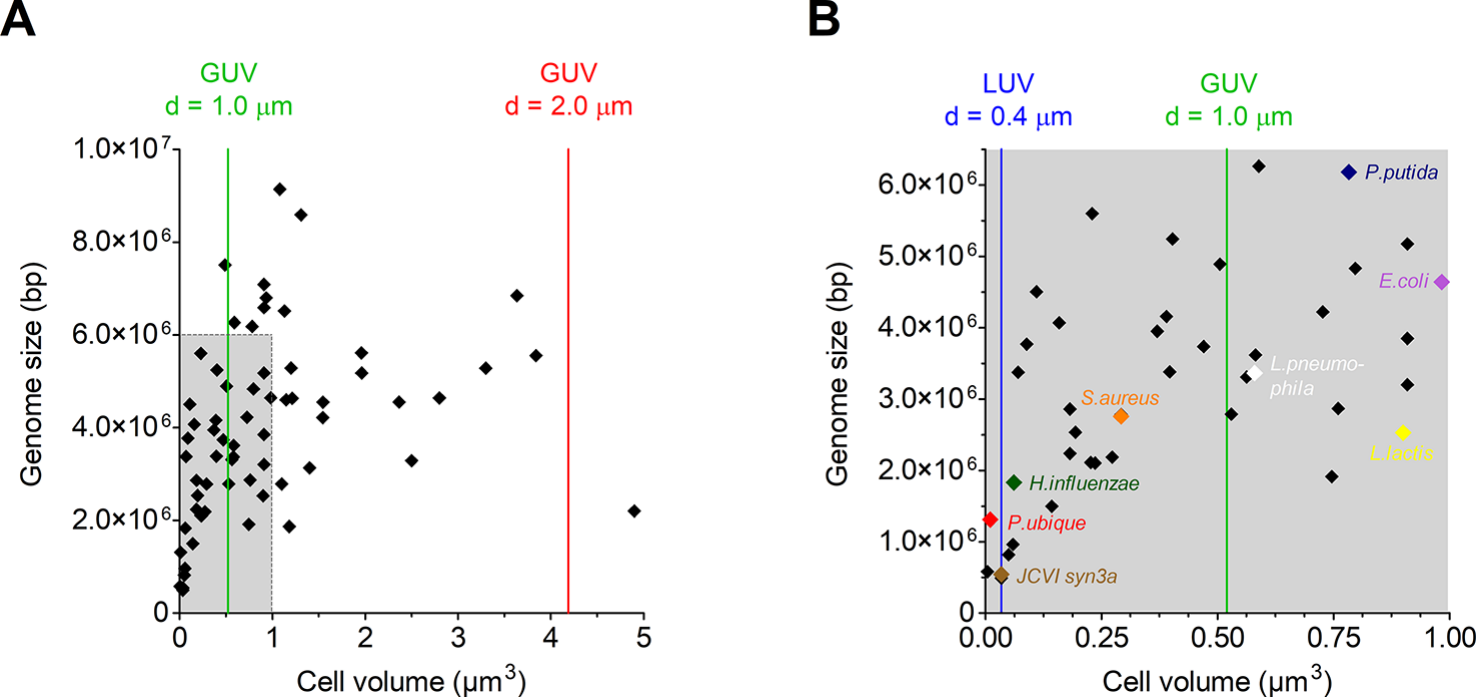
**Genome size as a function of cell volume**. The cell volumes of prokaryotes were obtained from ref (20). For each bacterial species, the respective genome sizes were collected from the NCBI Assembly database (Table S1). The blue line indicates the volume of an LUV with a diameter of 0.4 μm, while the green and red lines report volumes of GUVs of 1.0 and 2.0 μm in diameter, respectively. (A) Data for cell volumes up to 5 μm^3^. (B) Zoom-in of the data for cell volumes up to 1 μm^3^.

Synthetic cells that are to be built bottom-up from molecular building blocks can be designed by following the principle of compartmentalizing the metabolic machinery. This raises a question of what the optimal volume for such a compartment would be. Many bacteria have volumes of less than 1 μm^3^ and contain genomes with an upper limit in size of ∼6 Mbp (Figure 2a).^20^ The genome of JCVI-syn3a has a size of ∼0.5 Mbp^3,6^ and is enclosed in a volume of ∼0.03 μm^3^, similar to other pathogens (e.g., *Haemophilus influenzae*)^21^ but also to some free-living bacteria (e.g., *Pelagibacter ubique*)^22^ (Figure 2b). These volumes are comparable to that of large-unilamellar vesicles (LUVs) with a diameter of 400 nm, which suggests that a synthetic cell could exist out of an LUV equipped with a minimal genome of ∼0.5 Mbp. By contrast, a genome of 5 Mbp would require a volume of 0.5–4.2 μm^3^, that is, that of giant-unilamellar vesicles (GUVs) with a diameter of 1–2 μm.

To facilitate the construction of a complete synthetic metabolic network, it is arguably beneficial to work with a simpler set of metabolic modules that can be combined into a more complex system. These modules must be sufficiently simple to allow in-depth characterization of their kinetic behavior, which subsequently will facilitate the exploration of parameters that lead to emergent properties and functional designs that do not exist in nature. Modules that in our view are essential in any design and upon which a metabolic network can be built relate to the provision of energy and the establishment of ionic gradients along the membrane. Specifically, adenosine triphosphate (ATP) and nicotinamide adenine dinucleotides NAD((P)H) are the hub metabolites fueling life-like systems by provision of free energy and reducing equivalents;^23−25^ the ensemble of ionic fluxes across the bilayer generated by the action of membrane-embedded, ion translocating proteins is called ion motive force (IMF; or proton motive force, PMF, in the specific case of protons).^26^ The proton and sodium ion gradients are the main sources of electrochemical energy known in cells from all domains of life.^27^ Thus, a minimal cell-like system must efficiently incorporate simple pathways to utilize and regenerate ATP, NAD(P)H, and IMF, not only to drive the metabolism but also to maintain physicochemical homeostasis.

Here, we present a perspective and semiquantitative analysis of the requirements for the bottom-up construction of a basal metabolism suitable for synthetic cells, with a focus on the functions of the cell boundary. We propose that the compartment of the synthetic system should be selectively permeable to nutrients and waste products, and we give an overview of the lipid and protein components that should be included in a minimal membrane. We use JCVI-syn3a as a model organism to explore the vital functions of the energy supply, physicochemical homeostasis, transport of nutrients and waste products, and membrane growth. Lastly, we examine how different volumes would affect the relative protein surface area and lipid-to-protein mass ratios of the boundary and how these properties would reflect on the growth rate of the synthetic cells.

## 2 Compartmentalization: Lipid Vesicles

Membrane proteins have evolved to operate in a biological membrane and often exhibit dependencies on specific lipids for (optimal) functionality. Therefore, other vesicle-forming compounds, such as single chain-amphiphiles and block copolymers, generally do not or poorly support membrane protein function, with a few notable exceptions.^44−46^ Membrane transport proteins that undergo large conformational changes in the translocation step typically make essential interactions with, e.g., lipid head groups or require specific hydrophobic chains.^47−52^ Thus, if metabolism and energy conservation are to rely on multiple membrane proteins for selective communication with the external environment (Box 1), natural lipids are key building blocks to achieve compartmentalization for life-like entities. In addition, lipids can be synthesized using biosynthetic routes, which enable the required genetic encoding for a synthetic cell (see Section 3.4.1). Peptide-based membranes (e.g., formed of elastin-like peptides) have also been successfully used to demonstrate compartment growth and encapsulate biological reactions;^53,54^ the folding and insertion of a model membrane protein has also been achieved.^55^ Peptide-based membranes are genetically encodable and could be used in combination with lipids to tailor specific structural properties of the compartment. Alternative ways to concentrate molecules and generate confined compartments via phase separation are water-in-oil emulsions, coacervates, and membrane-less organelles^56,57^ where membrane proteins do not play a role. Biomolecular condensates are important for cellular and metabolic engineering, but these mechanisms of confinement are not discussed here.

### 2.1 Phospholipids

**Figure 3:**
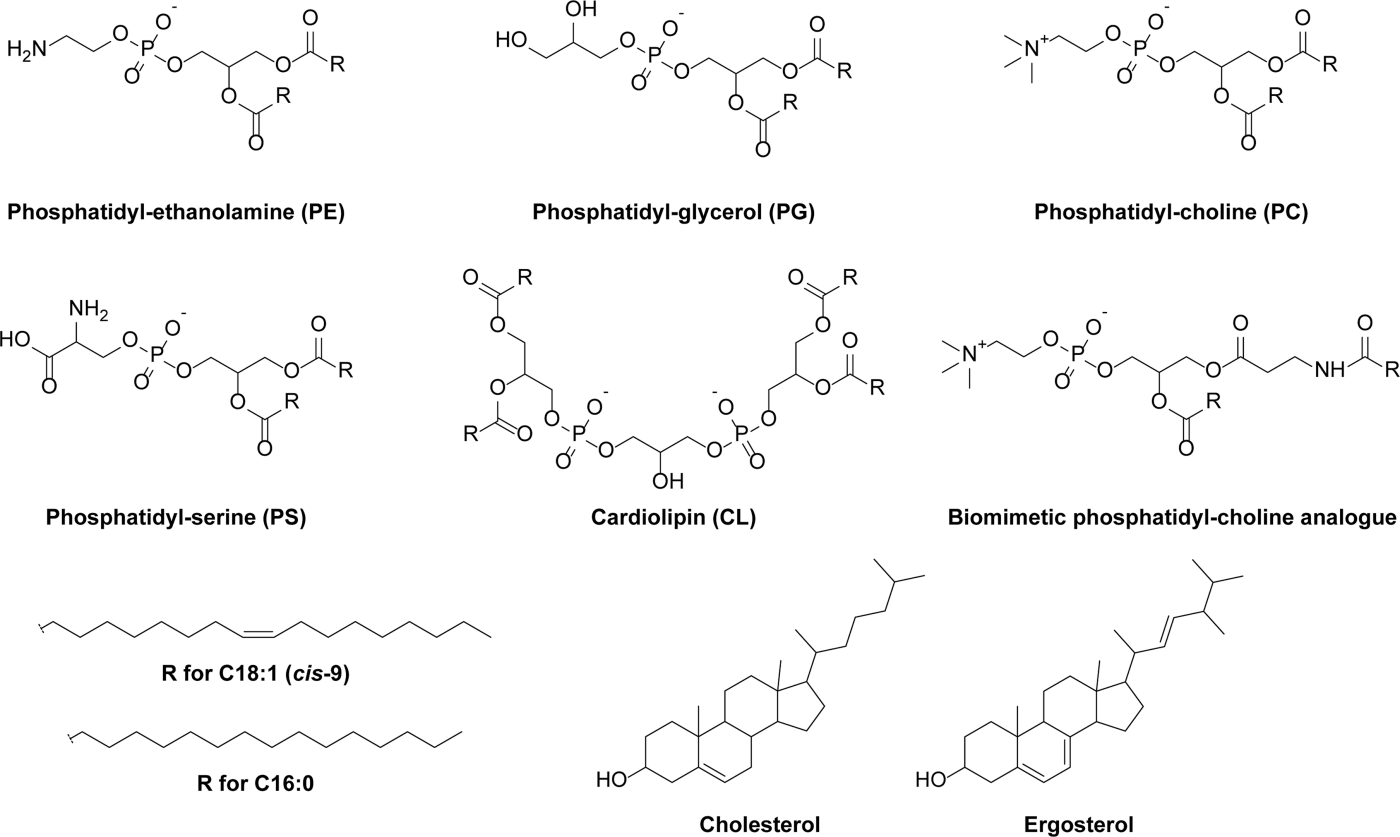
Lipid species are building blocks for synthetic cells. The biomimetic phosphatidylcholine analogue is from ref (99).

Selecting a lipid bilayer composition consisting of a minimal set of defined phospholipids that enable the functionality of a wide variety of membrane proteins is essential in building a synthetic cell (Figure 3). Phospholipid bilayers of differing complexity have been used to reconstitute purified membrane proteins.^47−52^ Complex mixtures extracted from natural sources, such as polar lipid extracts (e.g., from *E. coli*, soy, etc.), are also used, as their varied composition provides a close-to-native environment that meets the structure/activity requirements of many proteins. However, these mixtures do not easily enable us to determine the lipid properties minimally required for functionality, an important aspect of the building-to-understand approach.

On the other extreme are bilayers composed of single lipid species, such as the dioleoylphosphatidylcholine (DOPC) vesicles widely used in the field of biophysics (see, e.g., refs (43, 58−60)); these are also not suitable for developing minimal cell-like systems, as they do not support activity of many membrane proteins. For instance, a wide variety of membrane proteins studied to date require negatively charged phospholipids, such as phosphatidyl-glycerol (PG) or/and phosphatidyl-serine (PS), as well as the nonbilayer lipid phosphatidyl-ethanolamine (PE).^52,61,62^ The ratio of a bilayer to nonbilayer type of lipid is important for membrane formation and protein activity.^52^ We screened different synthetic phospholipid mixtures for the optimization of the activity of several membrane proteins. We have found that a minimal lipid mixture of PG/PE/PC (PC = phosphatidyl choline) at mole fractions of 25:25:50, supplemented with a sterol or specific lipid (see Section 2.2) supports the activity of many membrane proteins.

The acyl-chain composition of phospholipids directly influences membrane properties, such as its thickness, fluidity, and small solute permeability.^63^ Oleic (18:1 *cis*-9) and palmitic (16:0) acids are the most abundant acyl chains in living cells.^64−66^ We typically use dioleoyl or oleoyl-palmitoyl chains in our synthetic lipid mixtures.^48^ The global properties of lipid membranes such as lateral pressure in the headgroup and acyl chain region are important for the insertion and folding efficiency of (α-helical) membrane proteins, and these factors also have to be considered in the choice of lipid mixtures^67−70^ (Box 3).

Box 3 **Equipping phospholipid bilayers with membrane proteins: approaches and technical challenges**

**Membrane protein reconstitution in SUVs and LUVs.** Detergent-solubilized, purified membrane proteins can be reconstituted in SUVs and LUVs with lipid-to-protein ratios as low as 20:1 w/w, while maintaining their activity.^212^ In our hands, the reconstitution efficiencies typically vary between 40 and 70% at a lipid-to-protein mass ratio of 250:1 w/w and often (but not always) lead to a random orientation of the proteins in the membrane. This approach has the advantages that the quality of the membrane protein can be assessed prior to and after reconstitution, that the system is well-defined, and that complex membrane proteins with multiple transmembrane segments can be incorporated. While detergent-mediated protein reconstitution works well with minimal modules, increasing the system complexity brings about bottlenecks. In our attempts to compartmentalize the enzymes for phospholipid biosynthesis in LUVs, we have experienced the following technical limitations: (i) a requirement for high protein yields and purity levels, which are not trivial to obtain for certain membrane proteins; (ii) the difficulty of encapsulating peripheral membrane proteins, which tend to bind to the outer surface of the vesicles; and (iii) the low probability of distributing all components over the vesicle population, which leads to an increasing number of inactive vesicles (i.e., they lack one or more components of the pathway).

**Membrane protein reconstitution in GUVs.** The reconstitution of membrane proteins in GUVs currently poses a major challenge and requires the optimization of conventional formation methods or entirely new approaches. Most commonly used are gel-assisted swelling and electroformation.^228^ Integrin αIIbβ3, a membrane-anchored protein with a soluble active domain, has been reconstituted in GUVs by gel-assisted swelling.^229^ The voltage-gated ion channel KvaP has been reconstituted by gel-assisted swelling and electroformation.^230^ The latter approach has also been used to reconstitute a Ca^2+^-ATPase, the ABC transporter Opp,^231^ and bacteriorhodopsin.^232^ Solvent displacement is a less-trivial method for proteo-GUVs preparation, due to the use of organic solvents, although it was successfully adapted to reconstitute the integrin αIIbβ3 via the formation of water-in-oil droplets.^233^ To date, there is no general or robust protocol that allows the reconstitution of any complex, multispan transmembrane proteins (such as the membrane transporters) into GUVs of desired lipid composition.

**Transcription-translation in GUVs.** The in vitro synthesis of membrane proteins meets several requirements, including: (i) control over the orientation of the protein, unless it operates bidirectionally like solute/product antiporters; (ii) regulation of protein expression at the genetic level; and (iii) heredity of the molecular machinery, thereby avoiding dilution over daughter cells. Thus, externally fed, luminal synthesis of proteins is the route of choice toward the synthesis of autonomous cell-like systems that grow and divide. Due to the high molecular complexity of the protein synthesis machinery, transcription-translation in confinement is preferred in GUVs. Transcription-translation in confinement may be performed either by encapsulating cellular extracts^216^ or recombinant systems, such as the PURE,^218^ within vesicles. While cell extracts have a more complete formula and show higher expression yields,^217^ purified components are minimal yet highly defined and thus often preferred for the construction of synthetic cells. Both transcription-translation approaches also face technical challenges, including the difficulty of expressing functional ribosomes, which to date has only been achieved by coupling rRNA transcription to purified ribosomal proteins.^234^ In addition, the impaired ribosomal processivity poses a limit at the translational level.^235^

**Membrane insertion.** Finally, the in vitro transcription-translation of membrane proteins requires coupling to a mechanism (e.g., SecYEG/YidC or equivalent eukaryotic system) for the insertion and folding of functional membrane proteins. Some polytopic membrane proteins have been reported to self-insert in lipid bilayers in vitro without the aid of an insertion machinery such as the Sec translocon (reviewed in ref (71)). Examples are MraY, an enzyme responsible for cell wall synthesis,^67,236^ the β1-adrenergic receptor,^70^ the MscL mechanosensitive channel,^237^ and the lactose permease LacY.^69^ However, what is generally missing in these studies is a rigorous and quantitative analysis of the fraction of protein that is functionally incorporated in the membrane. In fact, the Sec system increases the efficiency of membrane insertion by lowering the energy barrier for a protein to enter the membrane. Thus, some protein may insert in the absence of Sec, but for a high efficiency of insertion and full functionality of the proteins the Sec translocon or equivalent machinery is needed. In one study, it is said that the bacterial SecYEG does not improve the in vitro membrane insertion efficiency of the membrane transport protein LeuT, but unfortunately the functionality of inserted LeuT has not been assessed. Moreover, the experiments have been performed in the absence of the signal recognition particle (SRP) and the SRP-receptor FtsY, two components required for SecYEG-dependent targeting and insertion. In our view, there is no compelling evidence for efficient and functional insertion in the lipid bilayer of polytopic membrane proteins in the absence of translocon and foldase components.

### 2.2 Other Lipids

Next to conventional phospholipids, other lipid species may be needed for a given membrane protein. For example, mitochondrial carriers have a dependency on cardiolipin (CL) for activity^72,73^ proteins derived from extremophiles may benefit from phosphoglycolipids,^74^ while the functionality of archaeal membrane proteins may depend on ether-type phospholipids.^75^ Nonpolar lipids such as sterols (e.g., cholesterol or ergosterol) can be required due to specific interactions with the proteins embedded in the membrane^47,76^ or their effect on the overall physicochemical properties of membranes (e.g., reduced passive ion permeability, modulation of membrane fluidity, and lipid dynamics and domain formation; for comprehensive reviews, see refs (77 and 78)).

Single-chain amphiphiles, e.g., fatty acids, can also self-assemble into bilayer structures when present above threshold concentration and within a certain pH window (centered around their p*K*_a_).^79−81^ Fatty acid vesicles are much more permeable to small polar molecules than phospholipid vesicles^82^ and can spontaneously undergo growth and division,^83−85^ which makes these amphiphiles particularly interesting for origin-of-life studies, given their prebiotic plausibility.^86,87^ The integrity of fatty acid vesicles is extremely sensitive to environmental physicochemical conditions; for example, low concentrations of divalent cations^88^ or pH changes^89^ can be detrimental to the bilayer stability. While their dynamic behavior was likely an advantage for the early onset of life on earth, it is undesirable for modern life that needs to maintain physicochemical homeostasis in a variety of environmental conditions. It is therefore not surprising that no known living organism relies on fatty acids alone for spatial confinement, although it is noteworthy that fatty acids and single-tail lipids can constitute a significant fraction of the amphiphiles that form the membrane of a cell.^90^

Fatty acids (and other single-chain amphiphiles) are the precursors of phospholipids. While it is possible to recapitulate isolated features of phospholipid vesicles with single-chain amphiphiles,^44^ it is evident that synthetic cells inspired by living organisms should not be composed exclusively of fatty acids. Rather, fatty acids should be supplied to (or internally formed within) synthetic cells as precursors for membrane expansion (Section 3.4.1). In this respect, studies focused on mixed single-chain amphiphiles/phospholipid vesicles^91,92^ are highly informative for the construction of synthetic cells^93^ and are necessary to obtain a better understanding of the bulk bilayer properties and the functional requirements of membrane proteins. For example, the conflicting data^94−98^ on whether flip-flop represents a rate-limiting step in the equilibration of free fatty acids across the membrane bilayer should be clarified.

Finally, it is important to note that each lipid type incorporated in a synthetic cell adds to the complexity of the system. Ultimately, a biogenesis/feeding mechanism is required for each lipid type present in the synthetic cell. Engineering biosynthetic pathways is a requirement for the autonomy of cell-like systems. It is thus important to have insight into the lipid requirements of the membrane proteins minimally needed to build a synthetic cell and, possibly, direct the choice of proteins toward orthologs with not too diverse lipid dependencies.

### 2.3 Size of Lipid Vesicles

Lipid vesicles span a large range of sizes, from small-unilamellar vesicles (SUVs, diameter <100 nm) to large-unilamellar vesicles (LUVs, diameter ∼100–1000 nm) and giant-unilamellar vesicles (GUVs, diameter >1000 nm).^100,101^ The size and volume of LUVs are in the same range as those of many microorganisms (Figure 2), while GUVs are typically the size of mammalian cells. The differently sized vesicles offer distinct advantages and limitations for the bottom-up construction of synthetic cells. For example, the reconstitution of membrane proteins in LUVs is well-established,^61^ for which these are preferred over GUVs. However, LUVs are affected by a large size distribution and are typically too small for optical microscopy observations. Instead, GUVs are large enough to allow for the direct visualization and identification of subpopulation behaviors.

#### 2.3.1 Preparation Methods

SUVs can be prepared by the hydration of a lipid film followed by sonication,^101^ which yields vesicles with a narrow size distribution.^102^ LUVs are then prepared by fusing preformed SUVs via freeze–thaw cycles, followed by an extrusion step to obtain more evenly size-distributed LUVs.^103^ Solvent displacement methods^101^ (such as reverse-phase evaporation) are also commonly used to produce LUVs,^104^ with the advantage of high encapsulation efficiency of soluble components and the possibility of forming asymmetric lipid bilayers.

GUVs can be prepared by gentle hydration,^105^ electroformation,^106,107^ and gel-assisted swelling.^108^ Various solvent displacement methods have been customized for GUVs formation, e.g., reverse-phase evaporation,^109^ inverted emulsions,^110^ and cDICE.^111^ Microfluidic tools are also widely used for the preparation of GUVs.^112^ The reconstitution of membrane proteins in GUVs poses a significant challenge.^101,113^ Currently, there is no robust technique to produce stable proteo-GUVs for any membrane protein. Methods are generally optimized for a certain membrane protein or synthetic cell module (Box 3).

#### 2.3.2 Encapsulation Capacity

Engineering an autonomous, functional synthetic cell will ultimately require many enzymes, each with a copy number of one or higher. From the data available for JCVI-syn3a, we argue that ∼500 genes will be needed for a bottom-up constructed cell. Accounting for membrane proteins (∼30% of the proteome) and the presence of multimers, this number reasonably translates to ∼250 soluble enzymes. Autonomy dictates that the metabolic machinery is eventually self-expressed from a genomic template. However, it is likely that initial engineering designs will have to rely on the encapsulation of pre-existing components due to the molecular complexity and technical limitations currently faced by in vitro transcription-translation systems (Box 3). In this context, it is important to determine how the encapsulation capacity of vesicles varies with respect to their size. The probability of encapsulating one or more molecules can be calculated as a function of the vesicle radius and of the protein concentration (Supporting Information Methods).

**Figure 4:**
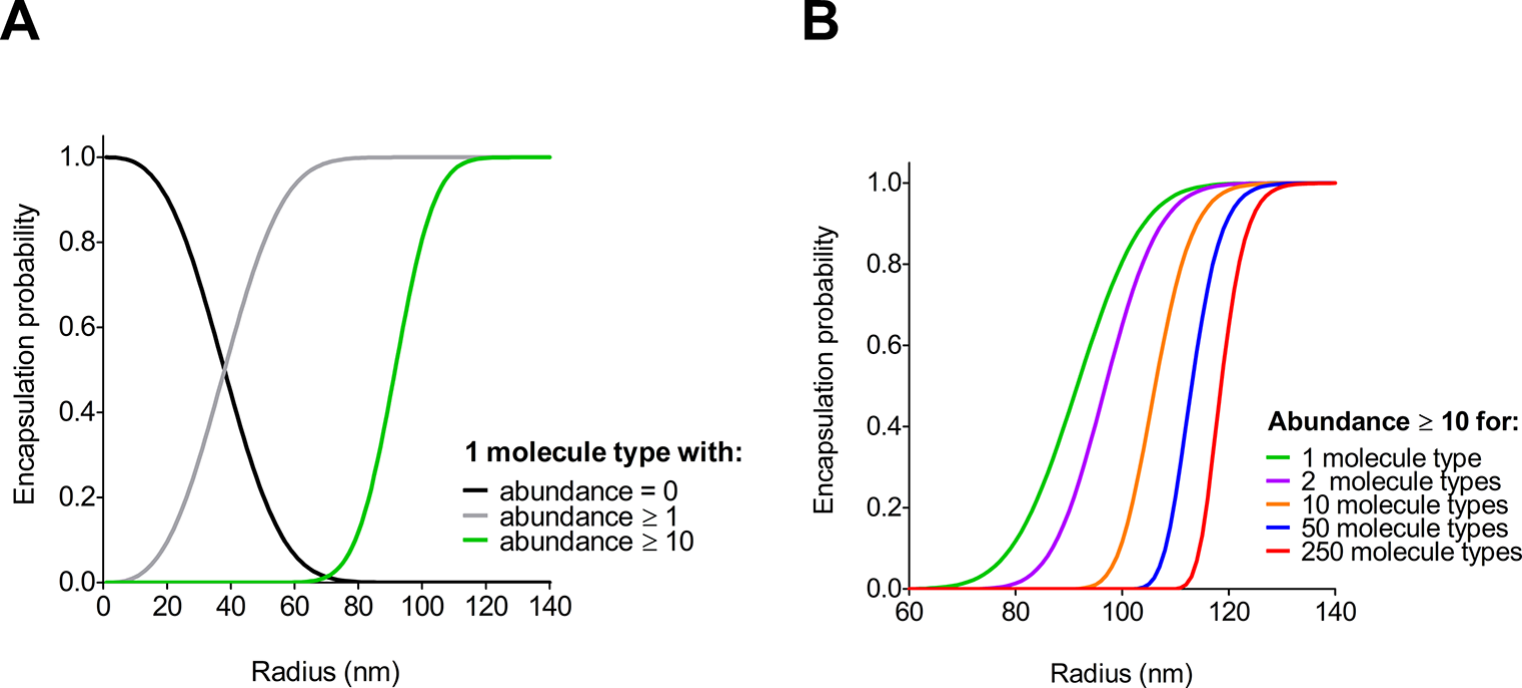
**Probabilities of encapsulating soluble components as a function of the vesicle radius.** The cumulative probabilities of a vesicle to contain one molecule with a certain (or larger) abundance were calculated from the Poisson probability mass function. The cumulative probability of a vesicle to contain multiple molecules was obtained from the independent probabilities of each molecule (see Supporting Information). (A) Probability of encapsulating one type of molecule at 5 μM concentration. Abundances (=0), (≥1), and (≥10) reflect the probability of finding zero, one (or more), and ten (or more) copies per vesicle. (B) Probability of encapsulating multiple types of molecules (1 to 250), each at 5 μM concentration and each with an abundance of 10 (or more) copies per vesicle.

Despite their relatively monodisperse size distribution, SUVs have an internal volume that is too small to accommodate sufficient amounts of enzymes for the construction of a synthetic cell, even at micromolar concentrations; hence, the encapsulation becomes stochastic, and most vesicles will be empty. A radius of at least 70 nm is required to ensure that each vesicle contains at least one or more copies of a given enzyme at 5 μM concentration, while a radius of 100 nm would ensure that 10 copies are minimally present (Figure 4a). To encapsulate a realistic number of enzymes (up to ∼250 different types, 5 μM each, at least 10 copies per type) requires LUVs with a radius of 130 nm or larger (Figure 4b, and our full analysis is shown in Figure S1). These estimations assume an infinitely small lipid bilayer, encapsulation efficiencies for each enzyme of 100%, and homogeneous size of the vesicle population. In practice, LUVs display a large size distribution,^63^ leading to a fraction of vesicles that does not contain all pathway components and is thus inactive.^42^ In addition, multiple proteins are likely to interact together, which will also have an effect on the encapsulation efficiency. Further, it is technically challenging to sufficiently concentrate multiple proteins to the required volume and encapsulate them without enormous loss of the precious purified components. Nevertheless, these estimations indicate that vesicles with a radius of ≥130 nm (within the range that is commonly employed for membrane reconstitutions) are large enough for the construction of life-like synthetic cells.

By contrast, GUVs have an internal volume sufficient to encapsulate complex molecular mixtures, circumventing stochasticity issues. In fact, cell-free transcription-translation machineries^38,114−118^ and components of the cytoskeleton^119−123^ have already been introduced into GUVs. In addition, GUVs also provide a larger membrane surface area to reconstitute membrane proteins once a suitable generic method has been developed. However, GUVs incur the cost of a less-favorable surface-to-volume ratio. This poses a serious problem for the delivery of large amounts of nutrients and other solutes by membrane transport proteins^124^ (see Section 4). Therefore, a compromise between LUVs and GUVs, that is, a small GUV with a diameter of 1–2 μm, might be ideal for the bottom-up construction of synthetic cells, as this would maintain a relatively large surface area and volume while minimizing the penalty associated with their ratio.

## 3 Membrane Modules for a Bottom-Up Minimal Metabolism

On the basis of the ATP requirements of an autonomous life-like system, Sikkema et al. have proposed a list of essential metabolic components.^62^ We classify these metabolites into two categories: recyclable cofactors (or coenzymes) and incorporated metabolites (or building blocks). Recyclable cofactors are metabolic intermediates that are responsible for the transfer of a functional group. These compounds transiently bind to the enzyme during the catalytic cycle and are subsequently reloaded with a new functional group. Net import (or synthesis) of such metabolites is not necessary as long as the cell does not grow. Examples of recyclable cofactors are phosphoryl donor ATP and electron donor NAD(P)H. The formulation of a specific cocktail of these recyclable metabolites is tightly interconnected with the ultimate metabolic reaction network design. We stress the importance of engineering recycling mechanisms in order to avoid systems reaching thermodynamic equilibrium.

Incorporated metabolites are the constituent units of macromolecules (e.g., amino acids and nucleotides) and lipids. These metabolites are sequestered from the cytoplasm and used by the cell to expand or replicate its own components before division. Therefore, building blocks should always be sourced from the external milieu. Prosthetic groups that are tightly bound to the enzymes are also regarded as building blocks.

**Figure 5:**
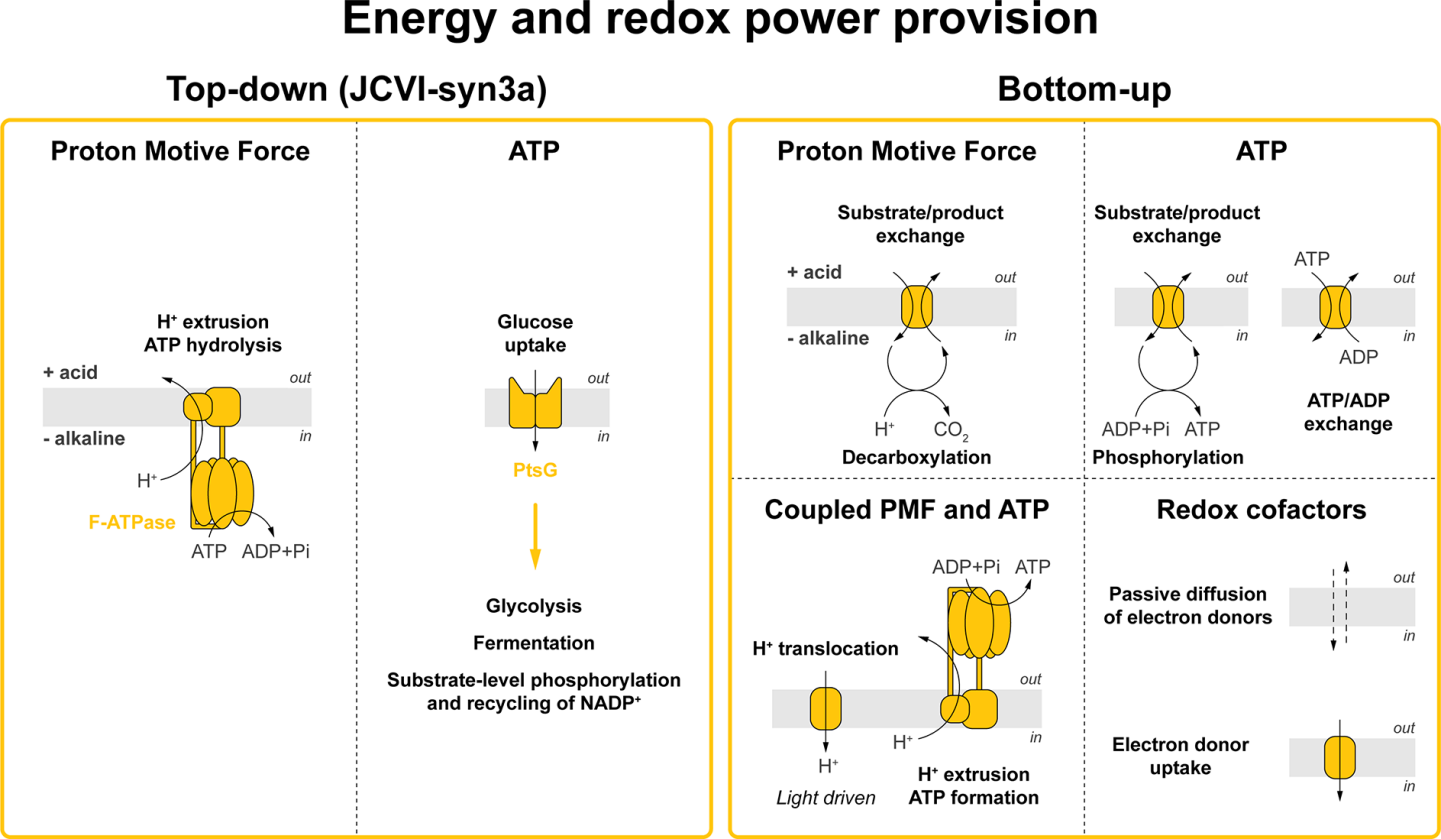
**Energy and redox power provision.** Membrane proteins used in the top-down approach and bottom-up designs of building minimal life-like systems. Left panel. Visualization of JCVI-syn3a membrane proteins annotated for energy conservation.^3,6,145^ Right panel. Overview of membrane proteins proposed for the engineering of energy and redox cofactor provision in bottom-up constructed synthetic cells. Reaction stoichiometries are not specified.

**Figure 6:**
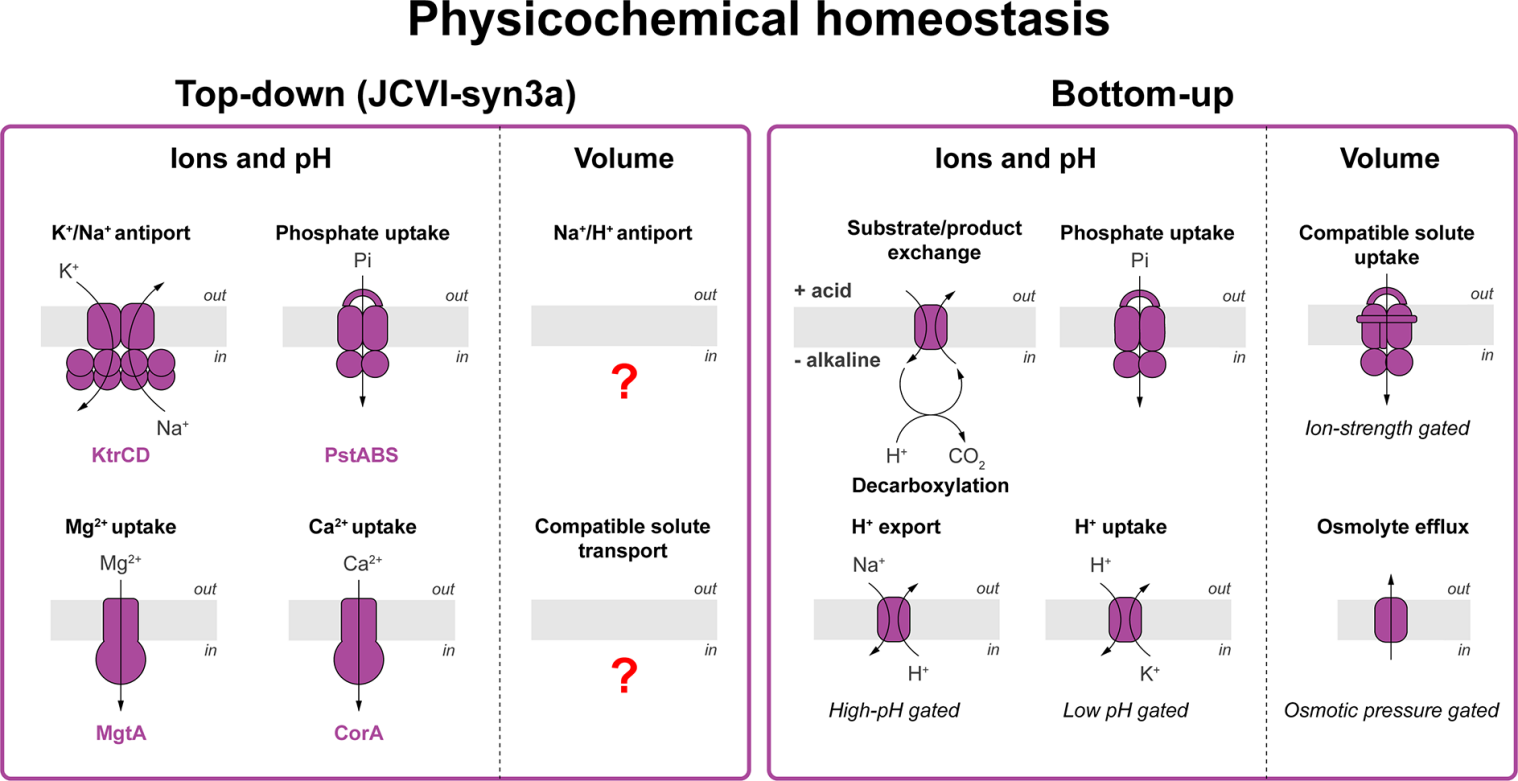
**Physicochemical homeostasis.** Membrane proteins used in the top-down approach and bottom-up designs of building minimal life-like systems. Left panel. Visualization of JCVI-syn3a membrane proteins annotated for physicochemical homeostasis.^3,6,145^ Right panel. Overview of membrane proteins proposed for the engineering of physicochemical homeostasis in bottom-up constructed synthetic cells. Reaction or transport stoichiometries are not shown.

**Figure 7:**
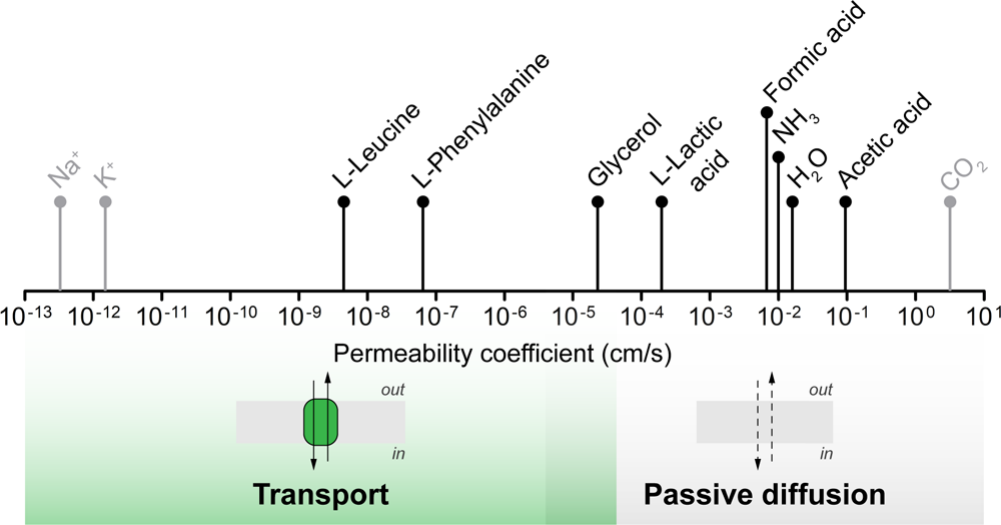
**Permeability coefficient of metabolites.** Permeability coefficients for amino acids, glycerol, weak acids, and water have been determined in vesicles composed of DOPC and POPC lipids at 20 °C.^143,144,173^ The permeability coefficient of ammonia was determined under identical conditions in vesicles composed of DOPE/DOPC/DOPG (50:12:38 molar).^63^ Permeability coefficients for sodium,^174^ potassium,^175^ and carbon dioxide^176^ were taken from different studies. For permeability coefficients lower than 1 × 10^–5^ cm/s, membrane transporters are arguably needed in synthetic systems, while compounds with higher permeability coefficients can rely on passive diffusion.

Based on the proposed classification, we identify several lipid bilayer-dependent modules that will be key for any design of a synthetic cell, namely: energy provision, physicochemical homeostasis, metabolite transport, and membrane expansion (Figures 5–7, Figure 9). We present a critical overview of the current state-of-the-art and designing principles for these modules, and we compare the corresponding metabolic reaction networks with those of JCVI-syn3a (Box 2).^3,6^

Box 2 **Membrane modules of the metabolism of JCVI-syn3a. The in silico metabolic reconstruction for JCVI-syn3a^3^ and the available functional annotations^6,145^ (Table S2) have been used. JCVI-syn3a genes are defined as essential, quasi-essential, or non-essential in agreement with ref (3).**

**Energy and redox power provision** The genome of JCVI-syn3a encodes the full glycolytic pathway. Glucose, mannose, and glucosamine are imported by PtsG, which is the membrane component of a PEP-dependent phosphotransferase system.^146^ The sugars are phosphorylated concomitantly with their transport and then metabolized. *N*-Acetylmannosamine is presumably taken up by an unknown ABC transporter and also metabolized into fructose-6-phosphate. The final product of glycolysis, pyruvate, is converted to acetate via the pyruvate dehydrogenase complex, phosphate acetyltransferase, and acetate kinase, yielding extra ATP. The genes for the F_o_F_1_-ATPase are present in JCVI-syn3a, allowing the generation of a PMF at the expenses of ATP. JCVI-syn3a synthesizes NAD^+^ and NADP^+^ via uptake of the precursor nicotinate (vide infra). NAD^+^ is reduced in the glycolytic pathway. Alternatively, a side shunt of glycolysis consisting of a nonphosphorylating glyceraldehyde 3-phosphate dehydrogenase (GapN) is responsible for NADP^+^ reduction.

**Physicochemical homeostasis.***Ion and pH homeostasis.* The genome of JCVI-syn3a encodes an ATP-consuming sodium/potassium antiporter (*ktrC*, *ktrD*), a P-type ATPase for magnesium uptake (*mgtA*), an ABC-importer for phosphate (*pstA*, *pstB*, *pstS*), and a putative magnesium/calcium transporter (*corA*). Sodium/proton antiport is assumed in the metabolic reconstruction; however, the corresponding gene(s) are not essential or quasi-essential. Yet, we consider one or more sodium/proton or potassium/proton antiporters essential for pH homeostasis. We note that a variety of transporters (e.g., for nucleosides and amino acids) are coupled to the PMF (vide infra).

*Osmotic pressure control.* Dedicated compatible solute importers are not annotated in JCVI-syn3a, and the metabolic model does not account for osmotic pressure regulation. In fact, *Mycoplasma* species do not possess a cell wall and most likely have little or no turgor.^147^ However, they still need to regulate their internal volume. Although the specific regulatory mechanisms are unknown, we argue that potassium ions and amino acids (e.g., l-glutamate) transport play a role in volume control in JCVI-syn3a.

**Metabolite transport.***Nucleotides.* A putative ABC transporter (*rnsA*, *rnsB*, *rnsC*, *rnsD*) may import all (deoxy)ribonucleosides, which are then phosphorylated by the kinases Tdk, Dak1, and Dak2 to yield the corresponding nucleotides. Nucleobase import is also included in the metabolic model with a proton symport reaction (albeit without gene assignation). The nucleobases can then be converted into nucleotides by several phosphoribosyl-transferases.

*Amino acids.* JCVI-syn3a imports oligopeptides by an ABC importer (*oppA*, *oppB*, *oppC*, *oppD*, and *oppF*), which are hydrolyzed by peptidases (*ietS* and others). A specific l-glutamate/l-aspartate transporter (*gltP*) ensures the uptake of l-glutamate, which is the most abundant amino acid in *Mycoplasma* species^148^ and possibly important for volume regulation (vide infra). In the metabolic model, l-glutamate and l-aspartate are taken up via a proton symport mechanism. In addition, two uncharacterized membrane proteins are modeled as putative unselective proton symporters for other amino acids.

*Vitamins and polyamines.* JCVI-syn3a encodes one energy-coupling factor (ECF) module^149^ (*ecfA1*, *ecfA2*, *ecfT*) and several uncharacterized substrate-binding subunits (S components, *ecfS1*, *ecfS2*, *ecfS4*) that have been assigned to the transport of folate, riboflavin, coenzyme A, nicotinate, and pyridoxal; an S component (*ecfS3*) responsible for the uptake of 5-formyl-THF is also included in the metabolic reconstruction. ABC-type importers for thiamine (*thiB*, *thiC*, and *thiQ*) and spermine (*potA*, *potB*, and *potC*) are quasi-essential in JCVI-syn3a.

*Waste export.* In JCVI-syn3a, several membrane proteins are annotated as ABC-type exporters. However, their functions are unknown.

**Membrane expansion.***Phospholipid biosynthesis.* Free fatty acids, cholesterol, and triacylglycerols are supplied by the hosts of *Mycoplasma* species,^150,151^ and, in turn, JCVI-syn3a relies on an exogenous feed of these components. The free fatty acids are phosphorylated in the cytosol by soluble fatty acid kinases (*fakA*, *fakB1*, and *fakB2*), followed by binding to the acyl-carrier protein (ACP) and transfer to glycerol 3-phosphate (*plsX*, *plsY*). Glycerol 3-phosphate is formed by phosphorylation of glycerol (*glpK*), which may passively permeate through the membrane.^144^ JCVI-syn3a encodes the pathway for phosphatidylglycerol (PG) and cardiolipin (CL) formation (*plsC*, *cdsA*, *pgpA*, and *pgsA* for PG, plus *clsA* for CL) but lacks enzymes for phosphatidylcholine (PC) and phosphatidylethanolamine (PE) biosynthesis. JCVI-syn3a also produces lipogalactan (*epsG*, *cps*) and Gal-DAG (*cps* and others).

*Membrane protein insertion.* JCVI-syn3a encodes the machinery for the cotranslational insertion of membrane proteins (*secA*, *secE*, *secY*, *secG,* s*ecD*, *secF*, and *yidC*), coupled to signal-recognition-particle docking (*ftsY*), quality-control (*ftsH*), and protein excretion (*lspA*). Other components involved in protein insertion/translocation in *E. coli* are missing in JCVI-syn3a (*secB*, *yajC*),^152^ but JCVI-syn3a may use other chaperone-like proteins to substitute for SecB, similar to *B. subtilis*.^153^

**Uncharacterized processes.** Despite the drastic genome reduction, JCVI-syn3a encodes 38 essential and quasi-essential membrane proteins for which it has not yet been possible to predict a function, that is, one-third of all membrane proteins; the same is true also for its soluble proteins. Computational efforts are ongoing to bridge the gap between these genes and the minimal proteome.^152^

### 3.1 Energy and Redox Power Provision

ATP is the main energy currency of living cells.^26^ Exponentially growing cells typically contain cytosolic ATP in the millimolar range. Importantly, upon hydrolysis, this cofactor is recycled, in order to continuously restore the energy pool. The importance of ATP recycling becomes evident by comparing the number of ATP equivalents present in a cell and the number of ATP equivalents required to sustain a full growth and division cycle. For example, JCVI-syn3a contains approximately 3.29 × 10^–3^ mmol/gDW ATP (Table 1), that is, 2.0 × 10^4^ molecules of ATP for a cell with a volume of 0.03 μm^3^, assuming a cell dry weight of 10.2 fg.^3^ However, JCVI-syn3a consumes a huge excess of ATP (46.58 mmol/gDW ATP or 2.9 × 10^8^ ATP equivalents) to sustain its metabolism throughout a cell cycle,^3^ which implies ∼10^4^ rounds of ATP recycling or a turnover of the cellular ATP pool of ∼1.5 s^–1^.

**Table 1:** **Main Cofactors in JCVI-syn3a and*****E. coli***^a^

**Cofactors**	**JCVI-syn3a**	***E. coli***
ATP	3.29 × 10^–3^ mmol/gDW (1.1 mM)	9.6 mM
ADP	n.a.	0.6 mM
GTP	2.19 × 10^–3^ mmol/gDW (0.7 mM)	4.9 mM
GDP	n.a.	0.7 mM
CTP	1.10 × 10^–3^ mmol/gDW (0.4 mM)	2.7 mM
CMP	n.a.	0.4 mM
NAD^+^	n.a.	2.6 mM
NADH	n.a.	83 μM
NADP^+^	1.05 × 10^–4^ mmol/gDW (40 μM)	2.1 μM
NADPH	n.a.	120 μM

a The cofactor concentrations were from ref (3) and from ref (133) for JCVI-syn3a and *E. coli*, respectively. For JCVI-syn3a, the cofactor concentration was converted from mmol/gDW into mM (in parentheses) by assuming a cell dry weight of 10.2 fg and an internal volume of 0.03 μm^3^ (both assumptions derived from ref (3)); n.a.: not available. We here refer to ATP, GTP, and CTP as recyclable cofactors, as they are regenerated in synthetic cells; in living cells they are also building blocks for nucleic acid synthesis.

The amount of ATP required to sustain the cellular metabolism scales with the molecular complexity and thus with the cellular volume. JCVI-syn3a has an internal volume of 0.03 μm^3^, comparable to that of LUV-sized synthetic cells. In agreement, it was calculated that 3.6 × 10^8^ ATP equivalents are needed for a vesicle of equal volume to undergo a full cell cycle,^62^ while *E. coli* (1 μm^3^) needs as much as 2.4 × 10^10^ ATP equivalents to sustain a cell cycle.^62^ A comparable energy requirement would be sufficient for a small GUV with a diameter of 1–2 μm. However, assuming that such a correlation would hold for larger volumes, it can be estimated that an average GUV with a diameter of 50 μm (65,500 μm^3^) would face a much higher energy demand (∼10^15^ ATP equivalents), which again points to small GUVs (or very large LUVs) as preferable compartments for bottom-up synthetic cells.

Besides ATP, electrochemical ion gradients (such as the proton motive force, PMF) play an essential role in energy provision. The magnitude and composition of the PMF vary for different cell types and environmental conditions, but typical PMF values range between −150 and −200 mV (−15.5 to −20.7 kJ/mol).^125^

The redox cofactors NADH and NADPH (Table 1) are hub metabolites involved in many energy-demanding processes and participate in over 1000 biochemical reactions.^126^ These redox cofactors play key roles in ATP and PMF (re)generation,^127^ protect cells against reactive oxygen species,^128^ function in cell signaling,^129^ and regulate gene expression and cell division.^130−132^ Numerous central metabolic pathways and cellular processes rely on the oxidation and reduction of redox cofactors as well as on the (re)generation of ATP and PMF. Consequently, a minimal out-of-equilibrium metabolic network should include modules for ATP, PMF, and redox cofactor (re)generation (Figure 5).

#### 3.1.1 Native Membrane Systems

Pathways for ATP, PMF, and redox cofactor (re)generation have been provided to in vitro systems by introducing organelles or crude membranes such thylakoids, chromatophores, and inverted membrane vesicles. For example, isolated thylakoid membranes have been encapsulated in microdroplets and used to drive the CO_2_-fixating CETCH cycle via NAD^+^ reduction and ATP regeneration.^134^ Likewise, chromatophores have been used to oxidize bacteriochlorophyll and reduce ubiquinone in GUVs, where the resulting PMF was used to synthesize ATP and drive mRNA synthesis.^135^ Similarly, inverted membrane vesicles have been used to generate a PMF via NADH oxidation and electron transport to oxygen and to synthesize ATP. In this system, the NADH oxidation activity was driving reactions of the pentose phosphate pathway, the Krebs cycle, and glycerol metabolism.

The use of such native microcompartments can provide the energy and redox cofactors for complex metabolic networks. However, they must be derived as fractions from living systems, have a complex and partly undefined molecular composition, and are not easily amenable for further engineering of cell-like systems. Therefore, better-defined, minimal modules are required for building a synthetic cell.

#### 3.1.2 Reconstituted Membrane Systems

The interconnection of ATP, PMF, and redox power provision in living cells has inspired the development of synthetic metabolic modules from purified and reconstituted components. For instance, an artificial photosynthetic organelle has been developed to generate a PMF and the subsequent synthesis of ATP upon illumination. The organelles were encapsulated within GUVs and used to power carbon fixation and actin polymerization^136^ as well as in vitro transcription-translation.^137^

A PMF has also been generated by NADH oxidation coupled to ubiquinone reduction with reconstituted mitochondrial complex I.^138^ In this system, ATP was produced externally and utilized to promote the cell-free expression of a reporter gene in the medium. The coreconstitution of a *bo*_3_ quinol oxidase and an ATP synthase led to the production of ATP by basic oxidative phosphorylation.^139^ These synthetic organelles have reported ATP synthesis rates^138,139^ that in some cases are comparable to that of *E. coli* F_o_F_1_-ATPase in membrane vesicles.^140^

#### 3.1.3 Reconstituted Metabolic Networks

As an alternative to redox- or light-driven PMF generation and ATP synthesis, reconstituted metabolic networks have been developed for energy conservation in LUVs. These metabolic modules are orthogonal to each other, thereby offering a high level of control over the synthetic reaction network. The l-arginine breakdown pathway has been reconstituted to generate ATP in LUVs and drive membrane transport to elicit partial volume and pH homeostasis.^42,63,141^l-Arginine breakdown only requires one membrane protein (ArcD), which imports l-arginine in exchange for l-ornithine (the end product of the pathway), plus three soluble enzymes (ArcA, ArcB, and ArcC); the formed CO_2_ and NH_3_ leave the vesicles by passive diffusion. Hence, the pathway runs for hours, away from equilibrium, and produces one ATP per l-arginine when ADP plus inorganic phosphate are supplied. The production of ATP has been used to drive compatible solute uptake^63^ and glycerol 3-phosphate synthesis.^42^ The current design of the l-arginine breakdown pathway produces ATP at a rate that would allow an LUV-sized synthetic cell^62^ to grow and undergo a complete cell cycle in about 10 h (Bailoni et al., manuscript in preparation). We note that a Pi importer is ultimately needed for sustained ATP formation by l-arginine breakdown, as part of phosphate will be incorporated in lipids and nucleic acids. In addition, a net uptake of ATP (or synthesis from precursors that are taken up as building blocks) is required under growth conditions to prevent dilution of the internal nucleotide pool over the daughter cells and, most importantly, to provide sufficient building blocks for the synthesis of nucleic acids (see Section 3.3.1).

Besides intraliposomal ATP formation, another complementary approach to supply synthetic cells with ATP relies on mitochondrial ATP/ADP carrier (AAC). This allows feeding of ATP from the outside to the synthetic cell in exchange for internal ADP (Heinen et al., manuscript in preparation). Such an exchange of ATP for ADP generates an inside-negative membrane potential and does not change the overall pool of available adenine nucleotides inside the synthetic cell.

Simple metabolic networks can also be used for the generation of a PMF. A wide variety of amino acids and dicarboxylic acids can be decarboxylated, and when the substrate is exchanged for the decarboxylated product, both a membrane potential (ΔΨ, inside negative) and pH gradient (ΔpH, inside alkaline) are formed. An example is the decarboxylation of L-malate^2–^ into L-lactate^1–^, as found in lactic acid bacteria.^142^ The uptake of L-malate^2–^ occurs in exchange for L-lactate^1–^, and the L-malate/L-lactate antiport thus generates a ΔΨ. A proton is consumed in the decarboxylation reaction inside the vesicles, hence the formation of a ΔpH. These pathways are advantageous for the modular design of a synthetic metabolism because the overall electrochemical gradient is formed independently of the pathway for ATP synthesis, in contrast to designs for energy conservation based on oxidative phosphorylation (vide infra). Metabolite decarboxylation can be used to drive the uptake of building blocks, such as amino acids and sugars, via secondary transporters (see Section 3.3.1). An overview of the different pathways for PMF and sodium motive force generation by substrate decarboxylation has been presented elsewhere.^62^

Pathways for the regeneration of reducing equivalents have also been designed. Formic acid is a convenient electron donor due to its low standard reduction potential (*E*′_0_ = −0.43 V)^25^ compared to nicotinamides (*E*′_0_ = −0.32 V) and its ability to permeate lipid membranes.^143,144^ In a minimal redox pathway, formate is utilized by a dehydrogenase that concomitantly reduces NAD^+^.^28^ The produced NADH can be coupled to NADP^+^ transhydrogenation, regenerating NAD^+^ and at the same time forming NADPH. This pathway for the regeneration of redox cofactors is functional in both LUVs and GUVs, and it has been used for NADPH-dependent conversion of glutathione disulfide into reduced glutathione, a known antioxidant that protects living cells against oxidative stress.^128^

### 3.2 Physicochemical Homeostasis

Living cells adapt to the constantly changing external milieu by maintaining their internal ion concentration, osmolality, and pH within viable physiological ranges, thereby achieving cellular homeostasis. Similarly, synthetic systems should also be equipped with reaction networks for the regulation of their internal physicochemical conditions (Figure 6).

#### 3.2.1 Ion Homeostasis

An essential property of living systems is that they can maintain ion gradients across their cellular membrane (see Section 3.1). For example, both JCVI-syn3a and *E. coli* accumulate potassium, chloride, and sodium ions in the tens to hundreds millimolar range (Table 2). Calcium and magnesium ions are present in lower amounts and mostly bound to macromolecules. Maintaining internal ion concentrations different from those in the external environment establishes electrochemical gradients across the membrane, an essential form of metabolic fuel (see Section 3.1). In JCVI-syn3a, several membrane transporters are dedicated to regulating the cytosolic ion levels (Box 2), and these will ultimately be required for any cell-like synthetic cell.

**Table 2:** **Main Ions in JCVI-syn3a and*****E. coli***^a^

**Ions**	**JCVI-syn3a**	***E. coli***
K^+^	0.840 mmol/gDW (286 mM)	30–300 mM
Na^+^	5.72 × 10^–2^ mmol/gDW (20 mM)	10 mM
Cl^–^	5.59 × 10^–2^ mmol/gDW (19 mM)	10–200 mM
HPO_4_^2–^	3.91 × 10^–2^ mmol/gDW (13 mM)	n.a.
Mg^2+^	7.76 × 10^–3^ mmol/gDW (3 mM)	30–100 mM
Ca^2+^	4.66 × 10^–3^ mmol/gDW (2 mM)	3 mM

a The ion concentrations were from ref (3) and from ref (154) for JCVI-syn3a and *E. coli*, respectively. For JCVI-syn3a, the ion concentration was converted from mmol/gDW into mM (in parentheses) by assuming a cell dry weight of 10.2 fg and an internal volume of 0.03 μm^3^ (both assumptions derived from ref (3)); n.a.: not available.

#### 3.2.2 Volume Homeostasis

Ion transport also plays a key role in the adaptation of cells to hypertonic stress. For example, potassium uptake is the primary response of many bacterial cells when they are confronted with an osmotic upshift of the medium. Subsequently, the K^+^ ions are replaced by neutral or zwitterionic solutes (so-called compatible solutes, e.g., trehalose and glycine betaine)^155^ in order to maintain their ionic strength within physiological limits. It is unclear how JCVI-syn3a operates the volume homeostasis (Box 2). A minimal system for adaptation to hypertonic stress is the ABC importer for glycine betaine OpuA, which is gated by ionic strength and equipped with a safety-check mechanism.^156^ Remarkably, OpuA provides partial volume regulation and pH homeostasis in vesicles equipped with the l-arginine breakdown pathway for ATP production^63^ (see Section 3.1.3). In the future, the osmotic stress response of synthetic cells could be expanded by introducing a protein that protects against hypotonic stress, such as a mechanosensitive channel.^157−160^ The mechanosensitive channels MscL and Pkd2 have been expressed by transcription-translation within GUVs and shown to be active under hypotonic conditions.^116,118,161^

#### 3.2.3 pH Homeostasis

In living cells, part of the internal buffering capacity comes from the protonatable groups of macromolecules (i.e., DNA, RNA, and proteins).^154^ However, control of the internal pH requires active mechanisms that directly or indirectly acidify or alkalinize the internal pH and keep it around neutrality. pH-sensing ion/proton antiporters allow a cell to rapidly respond to changes in the outside pH and prevent the cytoplasm from acidifying or alkalinizing.^162^ Also, simple metabolic networks activated by acidification, such as the L-malate decarboxylation pathway,^142^ increase the cytoplasmic pH by consuming a proton. The decarboxylation of amino acids such as l-glutamate and l-arginine has been shown to contribute to pH homeostasis in a variety of microorganisms^163,164^ (see also ref (62)). Bacterial amino acid decarboxylases have remarkably low pH optima,^165,166^ and their activity increases when the internal pH drops due to enhanced proton influx. Hence, the enzymes have a built-in self-regulatory mechanism to deal with lower internal pH values. Additionally, each system is coupled to an electrogenic substrate/decarboxylated product antiporter that generates a ΔΨ inside negative, and the overall pathways offer possibilities for metabolic energy conservation and pH homeostasis.

The proton-consuming l-arginine breakdown pathway from *Lactococcus lactis* has been reconstituted in a synthetic system with the aim to develop an ATP generation module (see Section 3.1.3).^63^ Intriguingly, this salvage pathway can cause internal acidification when the formed product of l-arginine deamination, L-citrulline, is not rapidly metabolized further, and a futile cycle with l-arginine/L-citrulline antiport follows the deamination. In this reaction, NH_4_^+^ ions are produced, and when NH_3_ leaves the vesicles by passive diffusion a proton is left behind. In synthetic vesicle systems, the internal pH drops when the production of ATP by l-arginine breakdown to l-ornithine exceeds the consumption of ATP by downstream pathways. Under these conditions, the futile l-arginine-L-citrulline cycle becomes dominant and acidifies the interior. This is an exciting example of emergent pathway behavior in reconstituted systems that has gone unnoticed in living cells. It should be possible to better control the pH in synthetic cells by combining the l-arginine breakdown pathway with a decarboxylation pathway.^62^ However, to gain full control of the internal pH one or more K^+^/H^+^ or Na^+^/H^+^ antiporters with different pH sensitivities and proton/ion stoichiometries will be needed.^167−169^ An effective pH regulatory transporter is NhaA of *E. coli*, which imports two protons in exchange for one sodium with a 1000-fold increase in turnover when the pH increases from 7.0 to 8.5.^170−172^

### 3.3 Metabolite Transport

**Figure 8:**
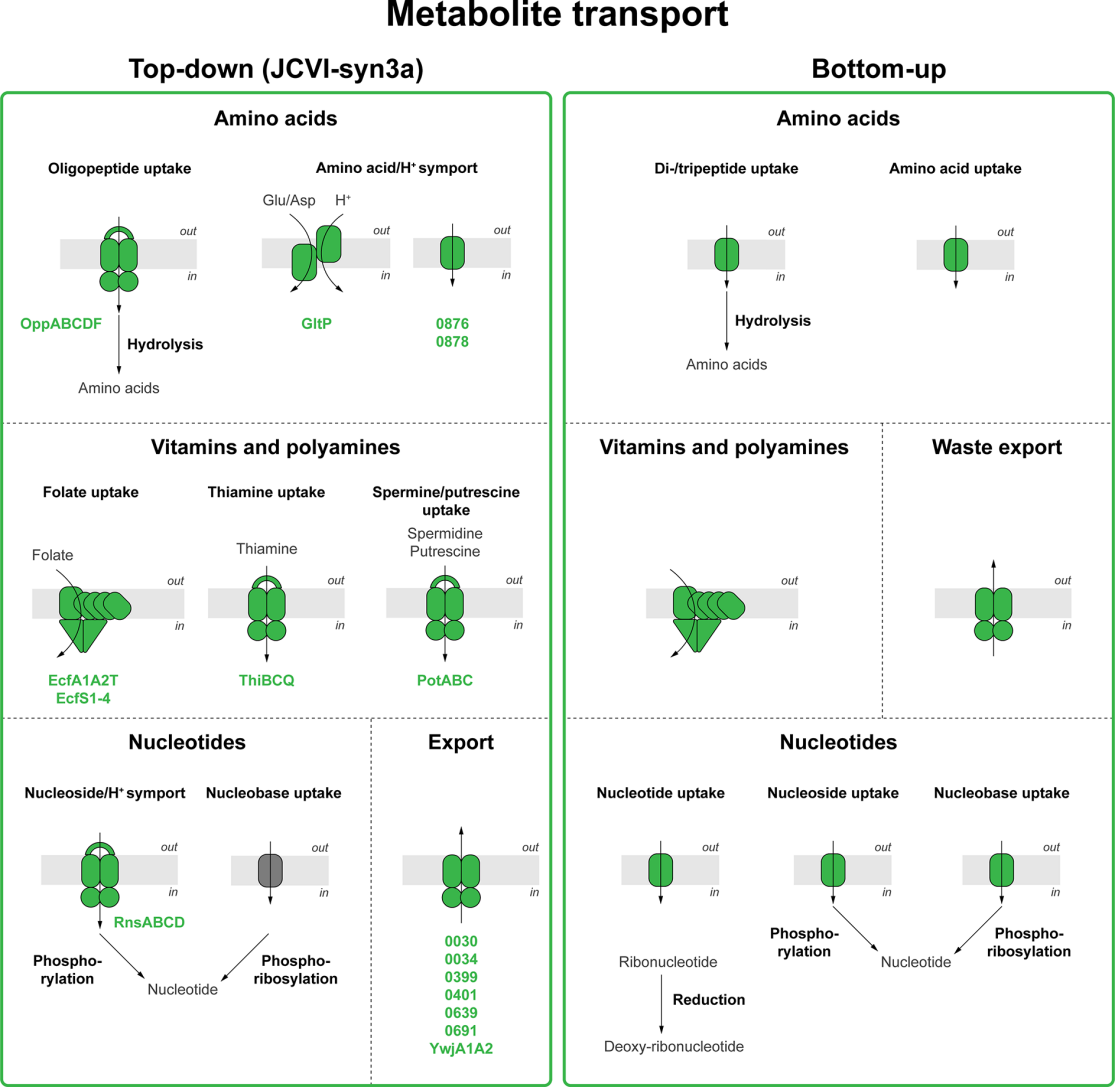
**Membrane transport.** Membrane proteins used in the top-down approach and bottom-up designs of minimal life-like systems. Left panel. Visualization of JCVI-syn3a membrane proteins annotated for membrane transport.^3,6,145^ Right panel. Overview of membrane proteins proposed for the engineering of membrane transport in bottom-up constructed synthetic cells. Although JCVI-syn3a uses, in many cases, ATP-driven transport systems, we envision that structurally simpler proton- or sodium coupled transporters could be used in the bottom-up constructed synthetic cell. Transport stoichiometries are not shown.

A requirement for synthetic cells to stay out-of-equilibrium is that substrates enter and waste products leave the system. In fact, when precursors are continuously supplied and end products do not accumulate, the metabolic reaction networks can, in principle, operate endlessly at a steady-state flux, provided the enzymes do not lose activity over time. In this regard, membrane permeability is a key property of cell membranes. Some valuable substrates (e.g., glycerol, weak acids) may enter the cell by simple diffusion through the lipid bilayer (Figure 7). Similarly, small neutral molecules such as carbon dioxide, oxygen, ammonia, and water can permeate membrane systems at a high rate.^173^ The diffusion of these molecules in membrane model systems poses generally no limitation for the reaction networks.^144^ However, lipid bilayers are relatively impermeable for ions (e.g., protons, potassium, sodium, calcium), sugars, most amino acids, vitamins, nucleotides, compatible solutes, and various metabolic end products.^144,173^ In these cases, membrane transport proteins are needed for import or export, either by facilitated diffusion or by active transport. Membrane transporters enable compartmentalized systems to maintain an internal chemical composition different from the external environment. They allow the accumulation of nutrients against their concentration gradients and generate electrochemical gradients for energy conservation and physicochemical homeostasis (Box 1). We present an overview of candidate transporters to equip synthetic cells with minimal modules for the acquisition of essential nutrients and the removal of metabolic end products (Figure 8).

#### 3.3.1 Uptake of Building Blocks: Nucleotides, Amino Acids, and Prosthetic Groups

The biosynthesis of macromolecules such as DNA, RNA, and proteins in living cells relies on the de novo synthesis of nucleotides and amino acids. However, these anabolic pathways are associated with high metabolic complexity and energy costs, which arguably should be avoided in the early stages of the development of synthetic cells. For nucleotide bioavailability, simpler routes are found in obligate intracellular parasites that have developed mechanisms for (deoxy)ribonucleobase and (deoxy)ribonucleoside import.^177−180^ Nucleobase and nucleoside import is retained by JCVI-syn3a (Box 2) and is arguably the simplest way to guarantee a complete pool of building blocks for DNA and RNA synthesis in synthetic systems together with dedicated cytosolic ribonucleoside kinases. The import of ribonucleotides has also been observed. Purine ribonucleotides are imported by parasites,^181−183^ while pyrimidine ribonucleotides are taken up by human^184^ and yeast mitochondria.^185,186^ Engineering nucleotide import would eliminate the need for internal phosphorylation (thereby easing the load on phosphate import) but would require a reductase^187^ and a suitable electron donor to produce the corresponding deoxy forms (see Section 3.1.3).

For amino acids, obligate parasites use transporters for extraction from their hosts,^188,189^ while oligopeptide import plays a pivotal role in the nutrition and communication of Gram-positive bacteria.^190^ JCVI-syn3a is also equipped with a mechanism for oligopeptide import (Box 2). Di/tripeptide transporters have been found in bacterial and mammalian cells, and in essence a single membrane protein can take up all amino acids if the proper mixture of di- or/and tripeptides is present in the medium. The relevant transporters belong the SLC15 family.^191−193^ They are high-capacity, low-selectivity transporters that are driven by the proton motive force. In combination with broad specificity aminopeptidase(s)^194^ that will rapidly convert the peptides into amino acids, it will be possible to deliver all 20 amino acids for protein synthesis. The significantly higher demand for certain amino acids (e.g., l-glutamate for cellular homeostasis) may be satisfied by tuning the peptide composition of the medium or by introducing additional selective amino acid transporters.

A milestone was set in the bottom-up synthesis of macromolecules by coupling self-encoded protein expression to DNA duplication in confinement.^114^ We envision that this system could be expanded in the future with selective building block uptake strategies, such as the ones proposed heretofore to keep macromolecule biosynthesis away from thermodynamic equilibrium.

Prosthetic groups are sequestered by proteins and are usually required in much lower concentrations than free metabolites. JCVI-syn3a has evolved a minimal system for the import of (the precursors of) vitamins and polyamines, consisting of a single transporter backbone (ECF module) coupled to a variety of highly specific substrate-binding subunits (S components)^149^ (Box 2). We argue that this approach could be recapitulated in a bottom-up system by equipping the synthetic cell with one ECF module and a selection of essential S components.

#### 3.3.2 Waste Export

Bacterial cells exploit export mechanisms to fulfill a plethora of functions, including the disposal of metabolic waste products, drugs, toxins, and signaling molecules. In a minimal synthetic cell, some export functions may not be readily required (e.g., for drugs and toxins), while others are beyond the focus of this perspective (e.g., cellular communication, recently reviewed^195^). However, a minimal cell-like system designed from scratch should also encompass export strategies for metabolic waste products that are membrane-impermeable. To this end, membrane antiporters that are selective for both the substrates and end products of a certain reaction network (e.g., ArcD in the l-arginine breakdown pathway; see Section 3.1.3) are particularly advantageous, as they combine the uptake of metabolic precursors with the removal of dead-end metabolites. In this way, a single protein couples functions that otherwise would require distinct membrane transporters.

### 3.4 Membrane Expansion: Phospholipid Biosynthesis and Membrane Protein Insertion

**Figure 9:**
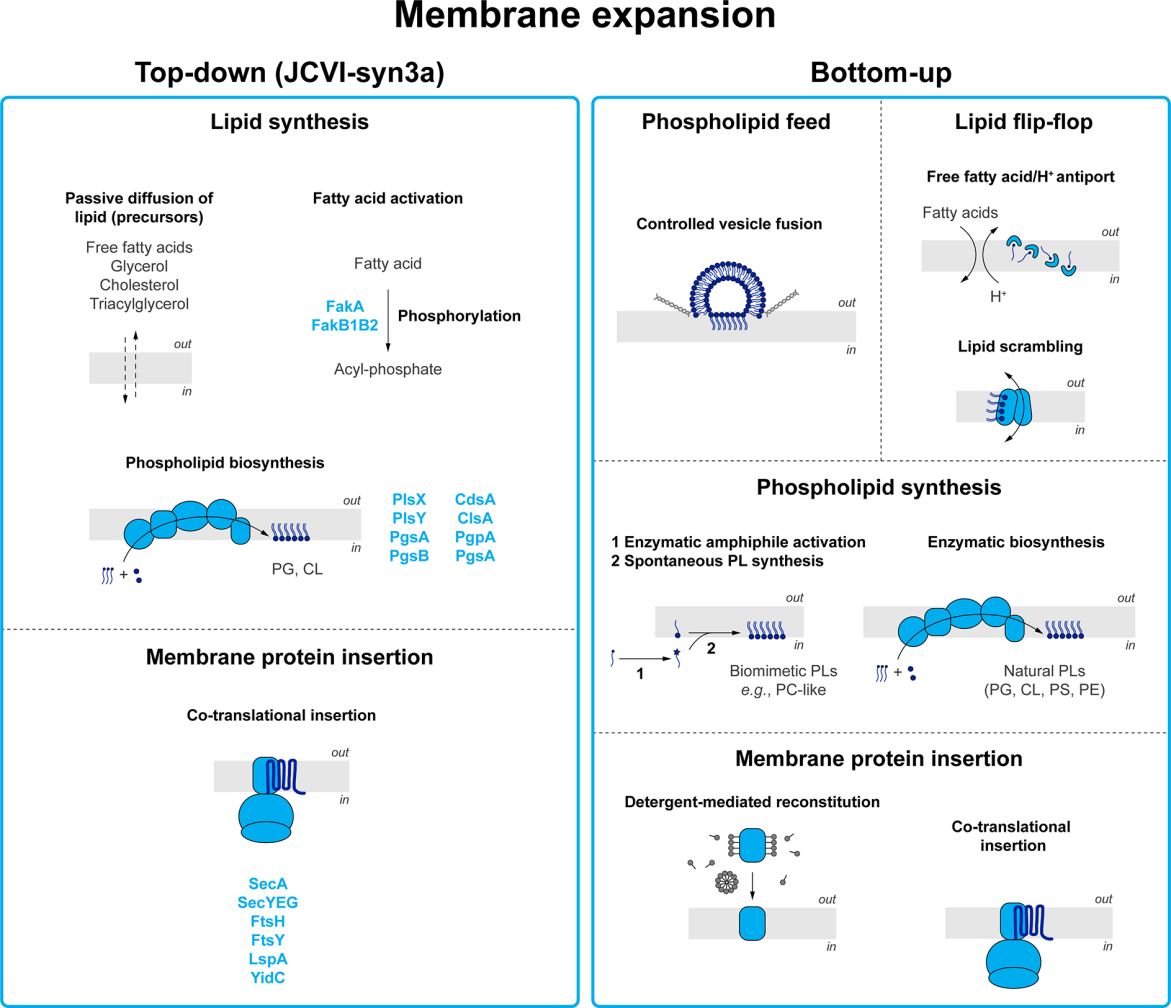
**Membrane expansion.** Membrane proteins used in the top-down approach and bottom-up designs of minimal life-like systems. Left panel. Visualization of JCVI-syn3a membrane proteins annotated for membrane expansion.^3,6,145^ Right panel. Overview of membrane proteins proposed for the engineering of membrane expansion in bottom-up constructed synthetic cells. Stoichiometries are not represented.

Besides the soluble nutrients, the components that constitute the lipid bilayer are essential building blocks of a synthetic cell. Thus, a minimal metabolism should also provide these in order for the system to ultimately expand its own boundary and divide^93,196,197^ (Figure 9).

#### 3.4.1 Phospholipid Biosynthesis

In living cells, phospholipid biosynthesis occurs via complex, multistep reaction cascades.^90,198,199^*Mycoplasma* species are able to take up phospholipids from the host organism,^151^ which is a capacity retained by JCVI-syn3a (Box 2). Analogously, it is also possible to directly grow synthetic cells by incorporating pre-existing phospholipids.^200^ For instance, controlled vesicle fusion guarantees that feeder vesicles fuse in a programmable manner with the synthetic cells, thereby delivering their building blocks to the membrane (phospholipids and membrane proteins) and to the cellular lumen (soluble components).^201^ Cargo release by controlled vesicle fusion has been successfully demonstrated in the presence of a variety of fusogenic compounds (e.g., SNARE peptide mimics,^202^ DNA tags,^203,204^ coiled-coil forming peptides,^205^ etc.), but it has not been assessed whether leakage occurs in the fusion process. Programmable vesicle fusion offers unique advantages compared to strategies that involve de novo synthesis of lipid. Feeding (phospho)lipids through pre-existing vesicles is fast and yields significant membrane expansion; the dispensability of protein expression for lipid biosynthesis and of actual lipid synthesis is an additional advantage. Hence, an overall optimization of the cellular resources, which can be repurposed toward other metabolic modules, can be envisioned. Next to membrane expansion, controlled fusion also brings the added bonus of feeding cytosolic nutrients and cellular components that are difficult to produce or recycle internally (e.g., ribosomes). However, in the absence of a mechanism to internally regulate the production and display of fusogenic tags, fusion-mediated membrane expansion remains dependent on external triggers, which may ultimately compromise the autonomy of synthetic life-like mimics.

Alternatively, the synthesis of phospholipids (or analogues thereof) has been explored through various approaches: (i) chemical, (ii) mixed chemo-enzymatic, and (iii) enzymatic. The chemical synthesis of phospholipid analogues has been achieved by linking an acyl-chain donor to a functionalized lysophospholipid by diverse reaction mechanisms, including click-chemistry, native chemical ligation, imine chemistry, transacylation, and others (see ref (206)), yielding biomimetic double-chain amphiphiles that retain the overall structure of natural phospholipids (with exception for an ester bond) and de novo self-assemble into membrane bilayers. An outstanding example includes the parallel self-regeneration of a catalyst, so that dilution was avoided and the catalytic process was sustained for a long time.^16^ We argue that membrane proteins incorporated in vesicles prepared from such biomimetic lipids will likely be functional,^207^ provided the appropriate headgroup composition is supplied (Section 2.1). However, the chemical approaches lack genetic control over the catalyst, making it ultimately difficult to autonomously regulate membrane expansion in cell-like systems with a genome.

A link to the genome may be established by mixed chemo-enzymatic approaches, where chemical reactions (i.e., spontaneous reactions or catalyzed by chemical catalysts) are coupled to biochemical ones (i.e., catalyzed by enzymes) to demonstrate de novo vesicle generation. For example, Bhattacharya et al. have developed a minimal synthetic pathway for lipid formation consisting of a water-soluble enzyme that activates a single chain amphiphile, followed by spontaneous acylation of a functionalized phospholipid precursor.^99^ Chemo-enzymatic phospholipid formation has also been coupled to the synthesis of the acyl-chain precursors by means of a type I fatty acid synthase.^208^ Beyond the advantage of eventually linking phospholipid formation to the regulation of gene expression, this route avoids the intrinsic molecular complexity of its multistep natural biosynthetic counterparts (vide infra). In addition, it provides a simple approach for phosphatidylcholine formation, which has not yet been realized in in vitro membrane model systems.

Finally, phospholipid formation can be achieved in vitro with a fully enzymatic approach by using (natural or engineered) multistep biosynthetic routes. With this approach, significant bilayer expansion was demonstrated by detergent-mediated reconstitution of the purified enzymes for PE and PG formation on the outer surface of small-unilamellar vesicles (SUVs). Despite its molecular complexity, this system is highly versatile. For instance, the PE and PG levels can be varied by changing the relative enzyme concentration, and different acyl-chain mixtures can be obtained by varying the pool of single-chain amphiphiles supplemented as substrates.^209^ Phospholipid biosynthesis has also been achieved in vitro by expressing PS biosynthetic enzymes within GUVs, albeit with modest yields.^38^ Genome-encoded phospholipid biosynthesis in confinement has in later studies been expanded by the inclusion of an acyl-chain formation module.^210^

A common bottleneck of all approaches that rely on long-chain fatty acids is the poor solubility of these and other bilayer-relevant amphiphiles in aqueous solutions. This poses an inherent limitation to phospholipid formation in nonleaky compartments, but it can be circumvented by provision of a continuous flow of diluted acyl-chain precursors.^42^ Furthermore, for sustainable lipid synthesis and membrane growth, lipid flip-flop mechanisms are required to move lipids from the *cis* to the *trans* side of the membrane (e.g., a lipid scramblase).^211^

#### 3.4.2 Membrane Protein Insertion

While a limited number of proteins, such as pore-forming toxins (Box 1), can spontaneously self-insert into model membranes, the vast majority of membrane proteins require membrane destabilization for their correct insertion into model membrane systems. Detergent-based protocols are available^61,212−214^ for the reconstitution of purified membrane components into LUVs (see also ref (215)). These approaches are convenient for studying simple synthetic modules in defined setups. However, the coreconstitution of multiple membrane proteins can become problematic in increasingly complex systems (Box 3).

Alternatively, membrane proteins can be produced in vitro by transcription-translation from a DNA template, exploiting either cellular extracts (e.g., from *E. coli*)^216,217^ or reconstituted cell-free translation systems (e.g., PURE).^218^ This approach is ultimately preferred over detergent-mediated reconstitution for autonomous cell-like systems. The PURE system has the advantage of being composed of well-defined components, and it has thus found wide application in bottom-up synthetic biology.^38,210,219^ However, membrane proteins synthesized in vitro by transcription-translation with PURE tend to display low activities,^38^ partly due to limitations intrinsic to PURE (Box 3) and partly due to the lack of control over protein insertion and folding. A number of studies have reported that polytopic membrane proteins self-insert into membrane bilayers when expressed cotranslationally, provided that specific phospholipid requirements are met.^68,69,220,221^ However, little or no quantitative information about the activity of the self-inserted membrane proteins is available (Box 3). Thus, the synthesis of membrane proteins in vitro should be inspired by living cells, which couple the translation of membrane proteins to their insertion (i.e., transertion) into the lipid bilayer. The cotranslational insertion of membrane proteins is primarily performed by the Sec/YidC machinery, a system conserved in archaea, bacteria, in the endoplasmic reticulum of eukaryotes (see also refs (222 and 223)), and in JCVI-syn3a (Box 2). The importance of membrane protein cotranslational insertion can be appreciated if one considers that roughly one-third of the entire proteome of living cells localizes at the cellular boundary.^154^

The Sec/YidC cotranslational insertion pathway has been studied in membrane systems prepared either via detergent-mediated reconstitution^224^ or expression from a DNA template.^225^ In vitro expressed SecYEG has been claimed to spontaneously self-embed into lipid bilayers, albeit with extremely poor efficiency.^225,226^ Therefore, we argue that catalytic amounts of Sec/YidC should be initially added to the membrane bilayer of the cell-like system (e.g., by detergent-mediated reconstitution) in order to facilitate the transertion of additional Sec/YidC and other membrane proteins.

### 3.5 Uncharacterized Processes

The genome of JCVI-syn3a encodes a significant portion of proteins with unknown functions^227^ (Box 2, Table S2), yet they are required for cellular growth, and their functions need to be unraveled.^3^ It is likely that these essential proteins will have consequences for the design of bottom-up constructed synthetic cells. In early engineering stages, a degree of uncertainty (use of components with unknown functions) will likely have to be tolerated in order to achieve complex functions such as membrane growth and division. Some evidence for this is already available. For example, certain membrane proteins display higher activities in membrane vesicles than in reconstituted vesicles of defined lipid compositions (Section 2.1). Most likely, specific lipid–protein interactions are missing in synthetic minimal membrane systems. Analogously, cell-derived translation systems guarantee much higher protein yields in comparison with their defined, cell-free counterparts like PURE (see Section 3.4.2, Box 3). The bottom-up assembly of cell-like systems may be ideal for uncovering missing factors and discovery of emerging properties, offering new insights for both bottom-up and top-down designs of synthetic cells.

## 4 What Membrane Surface Area Is Needed for a Minimal Metabolic Network?

The fluid mosaic model^238^ originally proposed to explain the physical properties of cellular membranes has been revised^239,240^ to account for membrane protein crowding, lipid phase domains, and skeletal structures.^241,242^ While the degree of crowding varies for different membrane types (i.e., the organism, cell, and organelle type) and cell cycle phases, membrane proteins generally occupy a large fraction of biological bilayers. Plasma membranes have a lipid-to-protein mass ratio of about 1:1 w/w, which corresponds to a surface occupancy by proteins of ∼25%.^243^

To what extent membrane and luminal macromolecular crowding are important for minimal life-like systems is an open question. The manipulation of membrane crowding is hindered by technical limitations in the reconstitution of membrane proteins. Physiologically relevant lipid-to-protein mass ratios have been achieved for structural characterization, but the activity of (transport) proteins decreases at low lipid-to-protein mass ratios.^244,245^ In order to obtain optimal protein functionality in LUVs, a lipid-to-protein mass ratio of at least 20:1 w/w is required.^61,212,246^ Protein insertion coupled to in vitro transcription-translation is to date also insufficient for creating low lipid-to-protein mass ratios (see Section 3.4.2, Box 3).

### 4.1 Membrane Surface Area Demand

Given the challenge to produce synthetic vesicles with physiological levels of protein crowding, the question arises whether, at relatively high lipid-to-protein mass ratios, such membranes can incorporate enough proteins to have sufficient capacity to transport all nutrients and other solutes needed for a minimal metabolism. This issue is particularly important for GUVs, as the surface-to-volume ratio is inversely proportional to the radius of the vesicles and membrane transport may become rate-limiting for luminal processes.^124^ Here, we narrow the problem down to estimating the smallest membrane surface area that would enable one to reconstitute membrane modules required for a minimal metabolism at a realistic lipid-to-protein mass ratio. To do so, we use the JCVI-syn3a annotated membrane functions (Table S2) as a proxy of what is needed in synthetic vesicles.

The total surface area occupied by membrane proteins can be estimated from the sum of their horizontal sections, each protein being approximated to a cylinder embedded perpendicularly in the membrane plane (Supporting Information Methods). The abundance of each protein was taken from the proteomic data of JCVI-syn3a in the exponential phase of growth (doubling time, *t*_d_ ≈ 2 h).^3,145^ This assumption is reasonable given that the total surface area of JCVI-syn3a (*r* = 200–250 nm)^3,247^ is comparable to that of LUVs extruded through 400 nm polycarbonate filters (*r*_average_ ≈ 230 nm).^63^ The relative protein surface area occupancy is the ratio between the total protein surface area and the total surface area of a sphere of a given radius.

**Figure 10:**
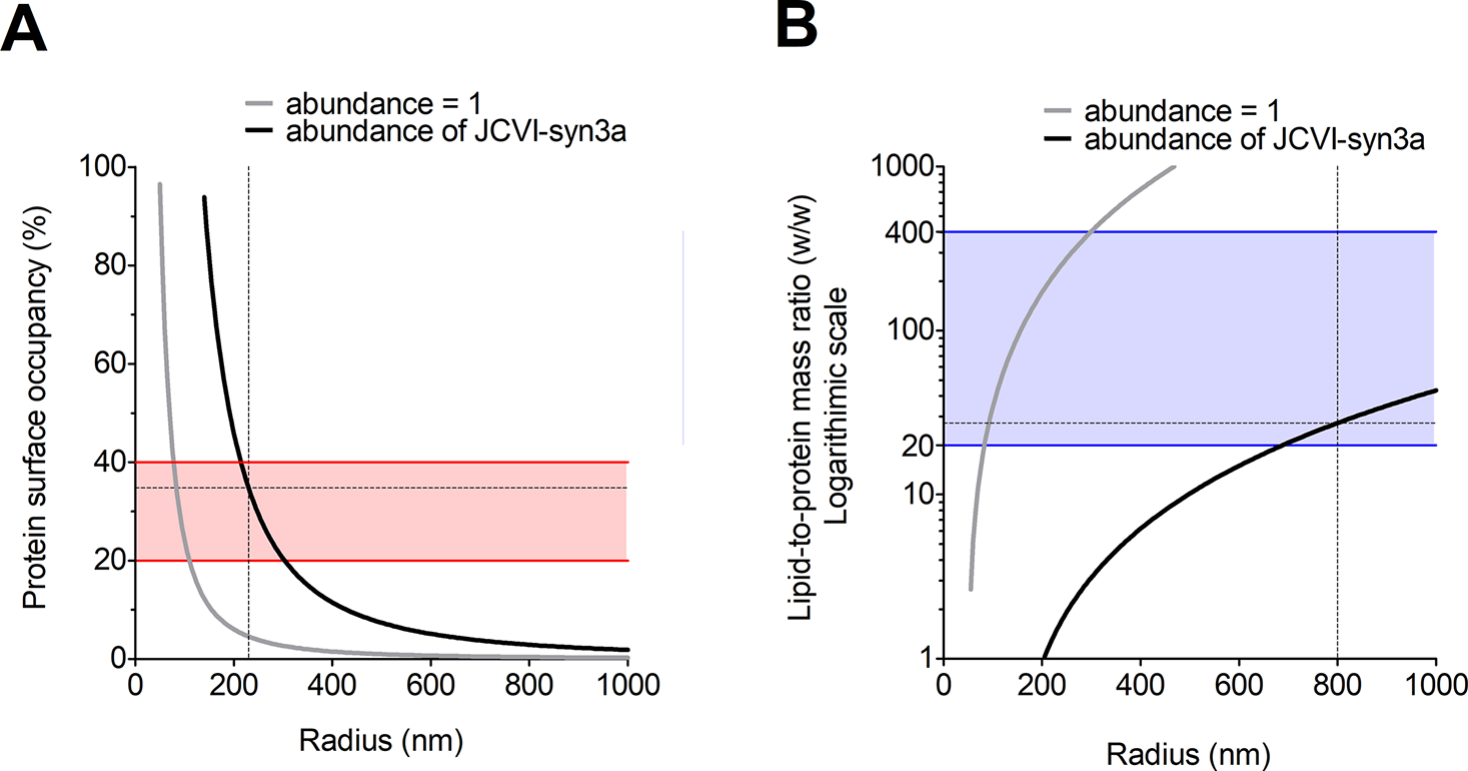
**Membrane surface area demand for a minimal metabolism in synthetic cells.** (A) Relative protein surface occupancy as a function of the vesicle radius. The relative protein surface occupancy represents the cumulative section area of the JCVI-syn3a membrane proteins. In one scenario, the JCVI-syn3a membrane protein abundance is taken into account (abundance of JCVI-syn3a, which corresponds to a doubling time of ∼2 h); the limit case where each protein is accounted for just once is also reported (abundance = 1). The physiological range of relative protein surface occupancies found in biological membranes is indicated (red shade).^243^ LUVs with *r*_average_ ≈ 230 nm fall in this range even if the protein abundance is taken into account (dashed black lines). (B) Lipid-to-protein mass ratio as a function of the vesicle radius. The total membrane protein mass was taken from JCVI-syn3a (abundance of JCVI-syn3a and doubling time of ∼2 h). The limit case of one copy number for each protein (abundance = 1) is also shown. A realistic range of lipid-to-protein mass ratios based on current technologies is shown (blue shade); the lower limit is imposed by technical limitations that affect reconstitution.^61^ LUVs with *r*_average_ ≈ 230 nm do not fall in the feasible range, and a radius >0.8 μm is required for a lipid-to-protein mass ratio of 20:1 w/w (dashed black lines).

Vesicles with radii smaller than 140 nm have a total surface area smaller than that of the proteins when the abundance of JCVI-syn3a is used (Figure 10a). Not only should the membrane proteins physically fit in the bilayer, also a certain amount of free lipid space is required. Further, a number of proteins interact peripherally with the membrane (e.g., enzymes involved in phospholipid biosynthesis, the ribosome for membrane protein insertion, etc.), so the relative protein surface area occupancy should probably not exceed the average values found in nature.^243^ Accounting for lipid occupancy would not represent a problem at the vesicle radii typically used for membrane protein reconstitution. A relative protein surface area of 35% would be achieved, if the JCVI membrane proteome was inserted in LUVs with *r*_average_ ≈ 230 nm.^63^

From the protein surface area occupancy, lipid-to-protein mass ratios can be estimated (Supporting Information Methods, Figure 10b). Vesicles with radii in the range of LUVs are too small to enable the reconstitution of the membrane proteins required for a minimal metabolism at realistic lipid-to-protein mass ratios and with a doubling time of ∼2 h. However, many bacteria that live in nature have much higher doubling times than JCVI-syn3a.^248,249^ It is likely that the initial designs of bottom-up cell-like systems will also have to compromise toward higher doubling times (Bailoni et al., manuscript in preparation). A higher doubling time may lower the required membrane protein abundance to values feasible in LUVs. For example, a doubling time of ∼50 h would lead to intermediate protein copy numbers that would enable >20:1 w/w lipid-to-protein mass ratios in LUVs with *r*_average_ ≈ 230 nm. This estimation assumes an inverse correlation between the protein abundance and the doubling time, which probably will not hold when the protein abundance becomes too low. Also, protein copy numbers lower than a certain threshold are not desirable due to stochasticity issues.

Vesicles with radii in the typical range of GUVs (10–50 μm) are sufficiently large for the reconstitution of JCVI-syn3a membrane proteins at physiological abundance and doubling time of ∼2 h, but vesicles with a radius of 1 μm, akin the size of bacterial cells like *E. coli*, also fall within the feasible range of lipid-to-protein mass ratios. Such bacteria-sized vesicles are particularly promising for the bottom-up engineering of life-like mimics, as they retain a relatively favorable surface area-to-volume ratio compared to regular GUVs and likely support sufficient transport capacity.^124^ We argue that bacteria-sized GUVs would allow for relatively fast growth at realistic levels of membrane proteins and encapsulated macromolecules. The corresponding volume range would also be ideal for the division protein machinery to operate, as it has been demonstrated that minimal Z rings self-assemble into rings of ∼1 μm diameter.^250^

## 5 Dynamics and Modeling

The components of the synthetic metabolic (sub)systems give rise to dynamic system-level behaviors, and computational modeling is key in understanding these emergent behaviors. Different types of modeling are conceivable, and some of them are outlined below and in Box 4.

Box 4 **Dynamic modeling of metabolism. A dynamic model of metabolism quantifies how the metabolite concentrations change over time. Building such a model requires two main steps. First, the model structure, i.e., the equations used to simulate the enzymatic and physicochemical processes, is developed. Second, the parameters of the model are set to the correct values**

**Model structure.** The model structure will largely consist of differential equations for each metabolite. These differential equations contain terms for each reaction in which a metabolite participates. The majority of these terms are enzymatic rate equations. These rate equations describe the rate of the (transport) reaction in question and may be a function of multiple metabolites (and other system properties, such as pH). Knowing the substrates and products of a reaction is not sufficient to obtain a rate equation, as this also depends on the mechanism (e.g., order in which substrates bind to the enzyme, conformational changes after binding). In addition to the mechanism itself, further assumptions about the kinetics can introduce or remove terms from the rate equation. For instance, it is often assumed that specific steps in the enzyme mechanisms are slower than other steps, which generally allows removal of some terms in the equation. For information about enzyme mechanisms and kinetics see refs (253−256), and for specific transport mechanisms see refs (257 and 258).

The choice of the rate equation for any reaction may significantly affect the reaction rate under certain conditions and, consequently, the dynamics of the system. Therefore, it is important to check whether the assumptions made about the mechanism hold under the conditions encountered in the actual system. This is not always the case with rate equations obtained from the literature. In fact, the equations from the characterization of enzymes or transporters often describe the initial conditions of a reaction. In these conditions, the buildup of, for instance, a reaction product or the membrane potential can be neglected, and they therefore do not appear as terms in the rate equation. Describing the full system dynamics goes beyond this initial state. If this is the case, extending the rate equations is often necessary. On the other end of the spectrum, there are equations that introduce a lot of parameters to the model, without essentially changing the observable system dynamics. For example, it may not always be necessary to explicitly account for all of the conformational states of a transporter. In short, selecting the correct rate equation requires knowledge of the system in which it is applied.

**Parameter values.** Parameter values can be taken from a database containing experimentally established enzyme parameters, for instance, Brenda,^259^ or they can be inferred from experimental data (either data available in the literature or a newly performed experiment) using a parameter estimation algorithm. Parameter values listed in a database are apparent parameter values. They are obtained under particular environmental conditions and assuming a particular rate equation. Rate equations in a dynamic model often differ from the rate equation used in characterization (see above). Consequently, a single apparent parameter is often a function of multiple parameters in the dynamic model.^260^ Therefore, care needs to be taken when incorporating database parameter values. For parameter estimation, both experimental data and a model are required. The result of parameter estimation is the set of parameter values that minimize the difference between the experimentally measured system variable and the simulated in silico system variable for the exact same conditions. For the intricacies of parameter estimation algorithms see refs (261 and 262).

In JCVI-syn3a, 155 genes (of a total of 493) encode gene products that catalyze 175 metabolic reactions. These metabolic reactions were first modeled^3^ stoichiometrically with a Flux Balance Analysis^251^ approach, which describes the metabolic fluxes at steady state. A second model^252^ extended the first to a dynamic model by assuming a rate equation (Box 4) for each metabolic reaction, thus obtaining an explicit description of how the system changes over time. This second model not only accounts for the dynamic nature of metabolism, but also describes the dynamics of other cellular processes that affect metabolism, including: transcription, translation, membrane growth, and diffusion of large particles. Simulations with this dynamic model yielded doubling times that are close to the experimentally observed doubling time of JCVI-syn3a. However, simulated dynamic profiles of system compounds, for instance, metabolite concentrations, have not been compared with respective experimental data. Deviations that may be found by comparing such profiles will likely be difficult to attribute to a specific mechanism or component due to the sheer complexity of this model.

A bottom-up constructed minimal metabolism may eventually be integrated with other key cellular functions, also resulting in a synthetic cell that is capable of growth and division. However, the bottom-up approach currently works with metabolic systems without any gene regulation. Despite their apparent simplicity, much mechanistic detail needs to be considered to provide a full dynamic description of such a system. For example, the l-arginine breakdown pathway^63^ imports l-arginine from the external environment and converts it in three enzymatic steps to ATP and waste products. The waste products (l-ornithine, ammonia, and CO_2_) leave the internal environment (by secondary active transport or passive diffusion).^63^ There are at least two system-level behaviors that emerge when the pathway operates. First, the pH of the internal environment changes due to proton consumption by the enzymatic reactions and passive diffusion of ammonia.^63^ A pH change could affect the enzymatic rates and the passive diffusion of protons across the membrane (due to the pH gradient that now exists with the external environment). Second, an unwanted transport reaction results in the buildup of a membrane potential. In the unwanted reaction, external l-arginine is not exchanged for l-ornithine (as was intended) but for the intermediate product L-citrulline. The exchange of l-arginine and l-ornithine is electroneutral, but the exchange of l-arginine for L-citrulline imports a net positive charge, consequently building up a membrane potential. The generated membrane potential in turn decreases the l-arginine/L-citrulline antiport.^63^ Thus, even in a simple bottom-up system, several effects, often of a physicochemical nature, on the system level can occur simultaneously. To ultimately understand the full dynamics of a bottom-up-built system, it is important to consider these system-level effects when integrating different metabolic modules, as they likely feed back onto other processes. For this reason, computational models are indispensable.

In our view, models of bottom-up systems have to be developed in a stepwise manner in tandem with the construction of the metabolic networks in the lab, starting with individual components and increasing the complexity of the system until all components are present. At each step the in silico simulations are compared to in vitro experiments with the same level of complexity. If there is a discrepancy between the two, then the model (and consequently our understanding of the system) is incomplete. Hypotheses that resolve these discrepancies can then be tested in silico and afterward verified experimentally. In contrast to modeling a large top-down developed system, modeling of a small bottom-up developed system yields system-level insights with mechanistic detail.^251^

## 6 Conclusions and Outlook

In this Perspective, JCVI-syn3a has been used as inspiration for the design of bottom-up constructed life-like synthetic cells. To operate a cell away from thermodynamic equilibrium, we reason that a boundary composed of lipids and selective membrane proteins is required. Hence, we provide a comprehensive description of the membrane modules minimally needed for sustainable metabolism and physicochemical homeostasis. We have evaluated the effect of the compartment size on the encapsulation efficiency and the surface area required for sufficient solute flux and other membrane protein functions. We conclude that bacteria-sized vesicles with a diameter of 1–2 μm are the most suited. We argue that the implementation of efficient protocols for the preparation of bacteria-sized vesicles is important. In parallel, robust in vitro transcription-translation and methods for membrane protein insertion need to be developed further. Such efforts will significantly advance the development of sustainable metabolic networks, which may ultimately lead to autonomous growth of synthetic cells.

## Supporting Information Available

Cell volumes and genome sizes of representative prokaryotes; Probabilities of encapsulating soluble components as a function of the vesicle radius, at varying concentrations and expected abundances; JCVI-syn3a membrane proteins involved in metabolic functions; Methods (PDF)

## Abbreviations

JCVI: J. Craig Venter Institute
*E. coli*: *Escherichia coli*
LUV: large unilamellar vesicle
GUV: giant unilamellar vesicle
Mbp: megabase pair
NCBI: National Center for Biotechnology Information
μm: micrometer
ATP: adenosine triphosphate
NAD(P)H: nicotinamide adenine dinucleotide (phosphate)
IMF: ion motive force
PMF: proton motive force
ClyA: cytolysin A
αHL: α-hemolysin
DNA: DNA
RNA: ribonucleic acid
DOPC: dioleoyl-phosphatidyl-choline
PG: phosphatidyl-glycerol
PS: phosphatidyl-serine
PE: phosphatidyl-ethanolamine
CL: cardiolipin
SUV: small unilamellar vesicle
cDICE: continuous droplet interface crossing encapsulation
FAD: oxidized flavin adenine dinucleotide
gDW: gram dry weight
ADP: adenosine diphosphate
GTP: guanosine triphosphate
GDP: guanosine diphosphate
CTP: cytidine triphosphate
CMP: cytidine monophosphate
NAD^+^: nicotinamide adenine dinucleotide oxidized
CO_2_: carbon dioxide
CETCH cycle: crotonyl-CoA/ethylmalonyl-CoA/hydroxybutyryl-CoA
mRNA: messenger ribonucleic acid
ArcA: l-arginine deiminase
ArcB: l-ornithine transcarbamoylase
ArcC: carbamate kinase
ArcD: l-arginine/l-ornithine exchanger
NH_3_: ammonia
Pi: inorganic phosphate
ΔΨ: membrane potential
NADP^+^: nicotinamide adenine dinucleotide phosphate oxidized
PtsG: phosphotransferase system glucose-specific component
PEP: phosphoenolpyruvate
ABC transporter: ATP-binding cassette transporter
GapN: glyceraldehyde 3-phosphat;e dehydrogenase
KtrC/D: potassium uptake proteins
MgtA: P-type ATPase for magnesium uptake
PstA/B/S: protein subunits of an ABC-importer for phosphate
CorA: putative magnesium/calcium transporter
RnsA/B/C/D: putative ribonucleoside ABC transporter
Tdk: thymidine kinase
Dak1/2: deoxyadenosine kinases
Opp(A/B/C/F): putative oligopeptide ABC importer
IetS: peptidase
GltP: glutamate/aspartate transporter
ECF(A1/A2/T) module: energy-coupling factor module
S-component: substrate-binding component
ThiB/C/Q: ABC-type importers for thiamine
PotA/B/C: ABC-type importers for spermine
FakA/B1/B2: fatty acid kinases
ACP: acyl-carrier protein
PlsY: glycerol-3-phosphate acyltransferase
PlsX: phosphate acyltransferase
GlpK: glycerol kinase
PlsC: 1-acyl-*sn*-glycerol-3-phosphate acyltransferase
CdsA: phosphatidate cytidylyltransferase
PgpA: phosphatidylglycerophosphatase A
PgsA: CDP-diacylglycerol-glycerol-3-phosphate 3-phosphatidyltransferase
ClsA: cardiolipin synthase A
EpsG: glycosyltransferase
Cps: glycosyl transferase
Gal-DAG: monogalactosyl-diacylglycerol
SecA/D/E/F/Y: protein translocase subunits
SecG: protein-export membrane protein
YidC: membrane protein insertase
FtsY: signal recognition particle receptor
FtsH: ATP-dependent zinc metalloprotease
LspA: lipoprotein signal peptidase
SecB: protein chaperone for export
YajC: Sec translocon accessory complex subunit
K^+^: potassium ion
Na^+^: sodium ion
Cl^–^: chloride ion
HPO_4_^2–^: phosphate ion
Mg^2+^: magnesium ion
Ca^2+^: calcium ion
OpuA: ABC importer for glycine betaine
NH_4_^+^: ammonium ion
NhaA: Na^+^/H^+^ antiporter
SLC15 family: solute carrier family of proton-coupled oligopeptide transporters
SNARE: soluble N-ethylmaleimide sensitive fusion protein attachment receptor
PURE: Protein synthesis Using Recombinant Elements
KvaP: voltage-gated potassium channel
rRNA: ribosomal ribonucleic acid
w/w: weight for weight
*t*_d_: doubling time
*r*: radius
