# Supplementary material for: Minimal Out-of-Equilibrium Metabolism for Synthetic Cells: A Membrane Perspective
Source: ACS Synth Biol. 2023 Apr 7;12(4):922–46. doi: 10.1021/acssynbio.3c00062 (PMC10127287; doi:10.1021/acssynbio.3c00062)
Supplement: Supplementary file 1 — sb3c00062_si_001.pdf [file sb3c00062_si_001.pdf]

Supporting Information

Minimal out-of-equilibrium metabolism for synthetic cells: a membrane perspective

Eleonora Bailoni[1], Michele Partipilo[1], Jelmer Coenradij[1], Douwe A. J. Grundel[2], Dirk J. Slotboom[1] and Bert Poolman[1]*

[1]Department of Biochemistry and [2]Molecular Systems Biology, Groningen Biomolecular Sciences and Biotechnology Institute, University of Groningen, Nijenborgh 4, 9747 AG, Groningen, The Netherlands

*To whom correspondence should be addressed (email: b.poolman@rug.nl)

Keywords: bottom-up synthetic cells | minimal metabolism | JCVI-syn3A | out-of-equilibrium | energy conservation | metabolite transport | membrane composition | physicochemical homeostasis

Table of contents:

Table S1. Cell volumes and genome sizes of representative prokaryotes

Figure S1. Probabilities of encapsulating soluble components as a function of the vesicle radius, at varying concentrations and expected abundances

Table S2. JCVI-syn3A membrane proteins involved in metabolic functions

Methods

Table S1. Cell volumes and genome sizes of representative prokaryotes. To avoid variability among different microbial subspecies, the genome size for each species was arbitrarily chosen from the NCBI Assembly database (links available in the 'Assembly' entry). The genome size is intended as the sum of the size of all chromosomes and plasmids, while chromosome size refers to the number of nucleotides of the main chromosome.

Species	Cell volume (μm^3)	Reference	Notes	Chromosome size (bp)	Genome size (bp)	Assembly
JCVI syn3a	0 .034	https://doi.org/10.7554/eLife.36842		543379	543379	ASM170832v2 - Genome - Assembly - NCBI (nih.gov)
Pelagibacter ubique	0 .010	https://doi.org/10.1038/nature00917		1308759	1308759	ASM1234v1 - Genome - Assembly - NCBI (nih.gov)
Acholeplasma laidlawii	0 .143	https://doi.org/10.1073/pnas.1514974112		1496992	1496992	ASM1878v1 - Genome - Assembly - NCBI (nih.gov)
Aerobacter aerogenes	3 .300	https://doi.org/10.1073/pnas.1514974112		5280350	5280350	ASM21574v1 - Genome - Assembly - NCBI (nih.gov)
Alteromonas haloplanktis/ Pseudoalteromonas haloplanktis	0 .909	https://doi.org/10.1073/pnas.1514974112	2 chr	3214944	3850272	ASM2608v1 - Genome - Assembly - NCBI (nih.gov)
Arthrobacter globiformis	0 .505	https://doi.org/10.1073/pnas.1514974112		4892409	4892409	ASM326117v1 - Genome - Assembly - NCBI (nih.gov)
Azospirillum brasilense	0 .909	https://doi.org/10.1073/pnas.1514974112	1 chr<br>5 plasmids	3005726	6587527	ASM131501v1 - Genome - Assembly - NCBI (nih.gov)
Azospirillum lipoferum	3 .636	https://doi.org/10.1073/pnas.1514974112	1 chr<br>6 plasmids	2988332	6846400	ASM28365v1 - Genome - Assembly - NCBI (nih.gov)
Azotobacter chroococcum	10 .909	https://doi.org/10.1073/pnas.1514974112	1 chr<br>6 plasmids	4591803	5192291	ASM81797v1 - Genome - Assembly - NCBI (nih.gov)
Bacillus cereus	3 .839	https://doi.org/10.1073/pnas.1514974112	1 chr<br>5 plasmids	5221581	5552031	ASM28367v1 - Genome - Assembly - NCBI (nih.gov)
Bacillus firmus	1 .149	https://doi.org/10.1073/pnas.1514974112		4597711	4597711	ASM984960v1 - Genome - Assembly - NCBI (nih.gov)
Bacillus licheniformis	0 .727	https://doi.org/10.1073/pnas.1514974112		4222597	4222597	ASM1164v1 - Genome - Assembly - NCBI (nih.gov)
Bacillus macerans	0 .909	https://doi.org/10.1073/pnas.1514974112		7085790	7085790	ASM972514v1 - Genome - Assembly - NCBI (nih.gov)
Bacillus megaterium	1 .960	https://doi.org/10.1073/pnas.1514974112	1 chr<br>7 plasmids	5066463	5610875	ASM200919v1 - Genome - Assembly - NCBI (nih.gov)
Bacillus stearothermophilus	0 .530	https://doi.org/10.1073/pnas.1514974112		2787229	2787229	GbsDonk1.0 - Genome - Assembly - NCBI (nih.gov)
Bacillus subtilis	1 .543	https://doi.org/10.1073/pnas.1514974112		4215606	4215606	ASM904v1 - Genome - Assembly - NCBI (nih.gov)
Bdellovibrio bacteriovorus	0 .089	https://doi.org/10.1073/pnas.1514974112		3769537	3769537	ASM220811v1 - Genome - Assembly - NCBI (nih.gov)
Beneckea/vibrio natriegens	1 .963	https://doi.org/10.1073/pnas.1514974112	2 chr	3248023	5175153	ASM145625v1 - Genome - Assembly - NCBI (nih.gov)
Clostridium butyricum	2 .800	https://doi.org/10.1073/pnas.1514974112	2 chr	3867296	4636588	ASM514508v1 - Genome - Assembly - NCBI (nih.gov)
Clostridium cellulolyticum	0 .159	https://doi.org/10.1073/pnas.1514974112		4068724	4068724	ASM2206v1 - Genome - Assembly - NCBI (nih.gov)
Clostridium thermocellum	0 .470	https://doi.org/10.1073/pnas.1514974112		3736353	3736353	ASM480395v1 - Genome - Assembly - NCBI (nih.gov)
Clostridium thermosaccharolyticum	1 .100	https://doi.org/10.1073/pnas.1514974112		2785752	2785752	ASM14561v1 - Genome - Assembly - NCBI (nih.gov)
Corynebacterium glutamicum	0 .563	https://doi.org/10.1073/pnas.1514974112		3309401	3309401	ASM1132v1 - Genome - Assembly - NCBI (nih.gov)
Delftia acidovorans	0 .933	https://doi.org/10.1073/pnas.1514974112		6796677	6796677	ASM1612741v1 - Genome - Assembly - NCBI (nih.gov)
Enterobacter aerogenes	1 .200	https://doi.org/10.1073/pnas.1514974112		5280350	5280350	ASM21574v1 - Genome - Assembly - NCBI (nih.gov)
Enterobacter cloacae	0 .230	https://doi.org/10.1073/pnas.1514974112	1 chr<br>2 plasmids	5314581	5598796	ASM2556v1 - Genome - Assembly - NCBI (nih.gov)
Enterococcus faecalis	0 .760	https://doi.org/10.1073/pnas.1514974112		2866948	2866948	ASM2286970v1 - Genome - Assembly - NCBI (nih.gov)
Escherichia coli	0 .983	https://doi.org/10.1073/pnas.1514974112		4641652	4641652	ASM584v2 - Genome - Assembly - NCBI (nih.gov)

Haemophilus influenzae	0 .061	https://doi.org/10.1073/pnas.1514974112		1830138	1830138	ASM2730v1 - Genome - Assembly - NCBI (nih.gov)
Lactobacillus bulgaricus	1 .182	https://doi.org/10.1073/pnas.1514974112		1864998	1864998	ASM5606v1 - Genome - Assembly - NCBI (nih.gov)
Lactobacillus casei	1 .400	https://doi.org/10.1073/pnas.1514974112		3132867	3132867	ASM219221v1 - Genome - Assembly - NCBI (nih.gov)
Lactobacillus plantarum	2 .500	https://doi.org/10.1073/pnas.1514974112		3285094	3285094	lactobacillus plantarum BFE5092 - Genome - Assembly - NCBI (nih.gov)
Lactococcus lactis	0 .900	https://doi.org/10.1073/pnas.1514974112		2529478	2529478	ASM942v1 - Genome - Assembly - NCBI (nih.gov)
Legionella pneumophila	0 .580	https://doi.org/10.1073/pnas.1514974112		3363998	3363998	ASM176633v1 - Genome - Assembly - NCBI (nih.gov)
Leptospira biflexa	0 .370	https://doi.org/10.1073/pnas.1514974112	1 chr<br>2 plasmids	3599677	3951448	ASM1768v1 - Genome - Assembly - NCBI (nih.gov)
Methylophilus methylotrophus	0 .182	https://doi.org/10.1073/pnas.1514974112		2860219	2860219	ASM37822v1 - Genome - Assembly - NCBI (nih.gov)
Mycoplasma genitalium	0 .004	https://doi.org/10.1073/pnas.1514974112		580076	580076	ASM2732v1 - Genome - Assembly - NCBI (nih.gov)
Mycoplasma pneumoniae	0 .050	https://doi.org/10.1073/pnas.1514974112		817207	817207	ASM127283v1 - Genome - Assembly - NCBI (nih.gov)
Mycoplasma pulmonis UAB CTIP	0 .059	https://doi.org/10.1073/pnas.1514974112		963879	963879	ASM19587v1 - Genome - Assembly - NCBI (nih.gov)
Myxococcus xanthus	1 .080	https://doi.org/10.1073/pnas.1514974112		9139763	9139763	ASM1268v1 - Genome - Assembly - NCBI (nih.gov)
Neisseria gonorrhoeae	0 .182	https://doi.org/10.1073/pnas.1514974112	2 chr	2232025	2236178	ASM2010v1 - Genome - Assembly - NCBI (nih.gov)
Neisseria meningitidis	0 .273	https://doi.org/10.1073/pnas.1514974112		2186098	2186098	https://www.ncbi.nlm.nih.gov/assembly/GCF_900638555.1
Paracoccus denitrificans	0 .403	https://doi.org/10.1073/pnas.1514974112	2 chr<br>1 plasmid	2858426	5242327	https://www.ncbi.nlm.nih.gov/assembly/GCF_004063735.1
Proteus vulgaris	0 .390	https://doi.org/10.1073/pnas.1514974112		4158443	4158443	https://www.ncbi.nlm.nih.gov/assembly/GCF_003812525.1
Pseudomonas aeruginosa	0 .589	https://doi.org/10.1073/pnas.1514974112		6264404	6264404	https://www.ncbi.nlm.nih.gov/assembly/GCF_000006765.1
Pseudomonas fluorescens	1 .130	https://doi.org/10.1073/pnas.1514974112		6515171	6515171	https://www.ncbi.nlm.nih.gov/assembly/GCF_900475215.1
Pseudomonas /vibrio natriegens	0 .909	https://doi.org/10.1073/pnas.1514974112	2 chr	3248023	5175153	https://www.ncbi.nlm.nih.gov/assembly/GCF_001456255.1
Pseudomonas perfectomarinus/ Stutzerimonas stutzeri	1 .545	https://doi.org/10.1073/pnas.1514974112		4545686	4545686	https://www.ncbi.nlm.nih.gov/assembly/GCF_019704535.1
Pseudomonas putida	0 .783	https://doi.org/10.1073/pnas.1514974112		6181873	6181873	https://www.ncbi.nlm.nih.gov/assembly/GCF_000007565.2
Rhizobium leguminosarum	0 .491	https://doi.org/10.1073/pnas.1514974112	1 chr<br>5 plasmids	5330343	7503296	https://www.ncbi.nlm.nih.gov/assembly/GCF_017357305.1
Rhodopseudomonas capsulata /Rhodobacter capsulatus	0 .582	https://doi.org/10.1073/pnas.1514974112		3616128	3616128	https://www.ncbi.nlm.nih.gov/assembly/GCF_014622665.1
Salinivibrio costicola	0 .397	https://doi.org/10.1073/pnas.1514974112		3381657	3381657	https://www.ncbi.nlm.nih.gov/assembly/GCF_001996185.1
Salmonella typhimurium	0 .797	https://doi.org/10.1073/pnas.1514974112	1 chr<br>1 plasmids	4793299	4831756	https://www.ncbi.nlm.nih.gov/assembly/GCF_000743055.1
Sphaerotilus natans	1 .214	https://doi.org/10.1073/pnas.1514974112		4630083	4630083	https://www.ncbi.nlm.nih.gov/assembly/GCF_900156335.1
Sphingopyxis alaskensis	0 .070	https://doi.org/10.1073/pnas.1514974112	2 chr	3345170	3373713	ASM1398v1 - Genome - Assembly - NCBI (nih.gov)

Staphylococcus aureus	0 .292	https://doi.org/10.1073/pnas.1514974112	2 chr	2755072	2782561	ASM609491v1 - Genome - Assembly - NCBI (nih.gov)
Staphylococcus epidermidis	0 .194	https://doi.org/10.1073/pnas.1514974112	1 chr<br>3 plasmids	2481008	2535366	ASM332573v1 - Genome - Assembly - NCBI (nih.gov)
Streptococcus faecalis	0 .909	https://doi.org/10.1073/pnas.1514974112		3201501	3201501	https://www.ncbi.nlm.nih.gov/assembly/GCF_006494875.1
Streptococcus pneumoniae	0 .227	https://doi.org/10.1073/pnas.1514974112		2110968	2110968	https://www.ncbi.nlm.nih.gov/assembly/GCF_001457635.1
Streptococcus pyogenes	0 .746	https://doi.org/10.1073/pnas.1514974112		1914862	1914862	https://www.ncbi.nlm.nih.gov/assembly/GCF_002055535.1
Streptococcus thermophilus	0 .236	https://doi.org/10.1073/pnas.1514974112		2102268	2102268	https://www.ncbi.nlm.nih.gov/assembly/GCF_010120595.1
Streptomyces coelicolor	1 .310	https://doi.org/10.1073/pnas.1514974112		8585093	8585093	https://www.ncbi.nlm.nih.gov/assembly/GCF_013317105.1
Thermoanaerobacterium thermosaccharolyticum	1 .100	https://doi.org/10.1073/pnas.1514974112		2785752	2785752	ASM14561v1 - Genome - Assembly - NCBI (nih.gov)
Vibrio anguillarum	2 .364	https://doi.org/10.1073/pnas.1514974112	1 chr<br>2 plasmids	3320860	4549571	ASM339957v2 - Genome - Assembly - NCBI (nih.gov)
Vibrio fischeri	0 .110	https://doi.org/10.1073/pnas.1514974112	2 chr	2970859	4503206	ASM2398347v1 - Genome - Assembly - NCBI (nih.gov)
Zymomonas mobilis	4 .900	https://doi.org/10.1073/pnas.1514974112	1 chr<br>4 plasmids	2058755	2200312	ASM305457v1 - Genome - Assembly - NCBI (nih.gov)
Nanoarchaeum equitans	0 .034	https://doi.org/10.1073/10.1038/417063a		490885	490885	https://www.ncbi.nlm.nih.gov/assembly/GCF_000008085.1/

Figure S1. Probabilities of encapsulating soluble components as a function of the vesicle radius. Three different concentrations (1, 5 and 10 μM) of soluble components were analyzed. The vesicle radius required for a given encapsulation probability varies reciprocally with the concentration. By contrast, the vesicle radius correlates with the expected abundance, that is, larger vesicles are required to obtain higher abundance values at a given concentration.

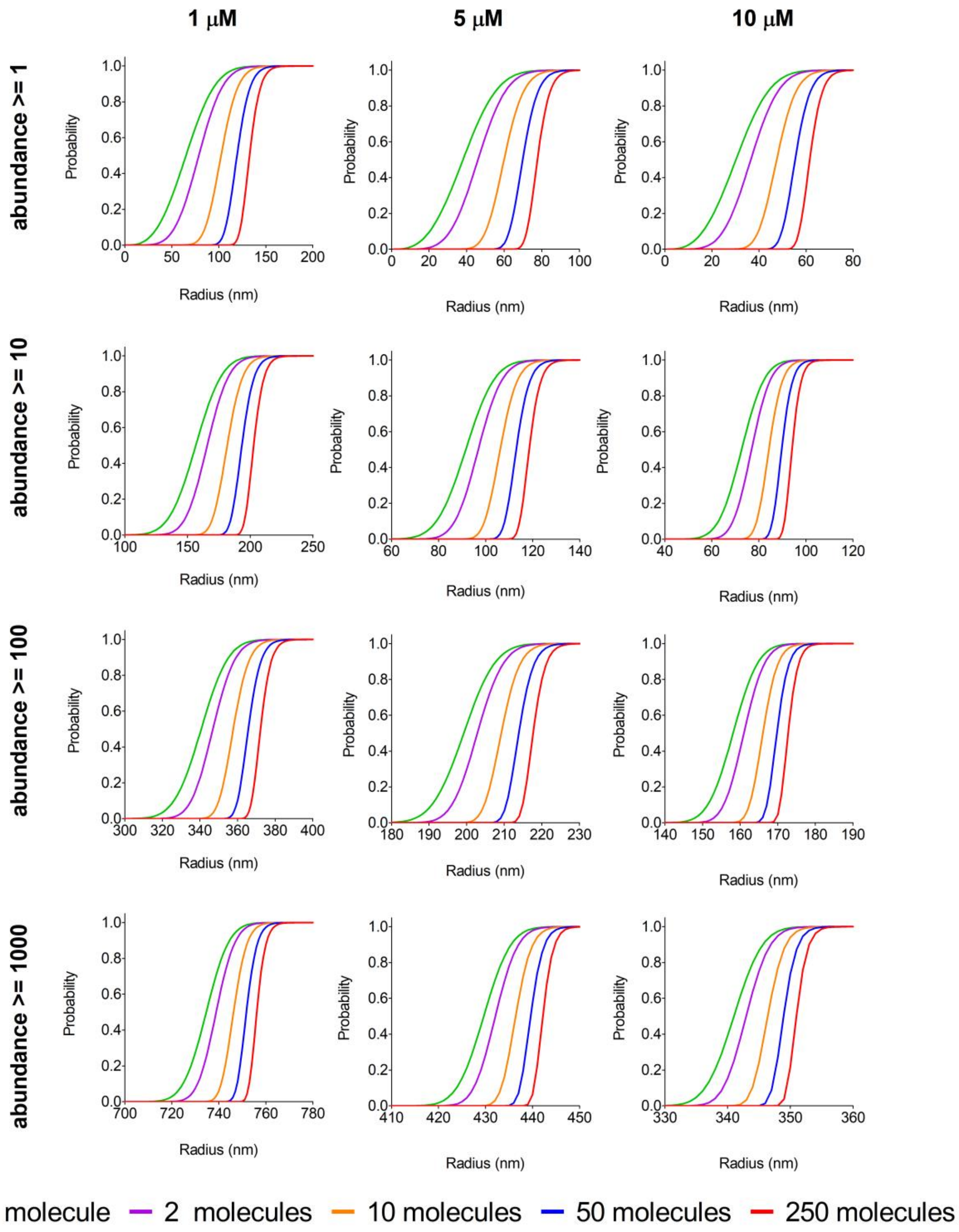

Table S2. JCVI-syn3A membrane proteins involved in metabolic functions. Information retrieved from[1]. MW=molecular weight; NA=not available; PDB[2]; AlphaFold[3,4].

Function	Gene	MW (kDa)	Copy Number	Structure ID	Reference
Energy generation	*ptsG*	80.7	831	6BVG	PDB
	atpA	58.2	327	6Q45	PDB
	atpB	33.0	9	6FKF	PDB
	atpC	11.2	109	6Q45	PDB
	atpD	52.3	347	6Q45	PDB
	atpE	10.2	NA	4BEM	PDB
	atpF	20.5	181	Q6MS90	AlphaFold
	atpG	31.7	137	6Q45	PDB
	atpH	20.6	32	6N2Y	PDB
Ion transport	*ktrC*	27.4	306	6I8V	PDB
	ktrD	60.3	18	4J7C	PDB
	mgtA	105.9	65	1MHS	PDB
	pstA	78.3	27	Q6MTC2	AlphaFold
	pstB	30.5	123	4U00	PDB
	pstS	43.7	56	4Q8R	PDB
	corA	100.9	81	1MHS	PDB
Cofactor uptake	*ecfA1*	46.2	NA	4HUQ	PDB
	ecfA2	34.4	274	4HUQ	PDB
	ecfT	37.9	25	4HZU	PDB
	ecfS1	27.2	150	Q6MTM7	AlphaFold
	ecfS2	34.0	10	Q6MTJ9	AlphaFold
	ecfS3	25.0	1	4HUQ	PDB
	ecfS4	34.7	1	Q6MS24	AlphaFold
	thiB	68.2	2	Q6MSI6	AlphaFold
	thiC	54.7	211	3E78	PDB
	thiQ	28.9	41	5LJ9	PDB
	potA	40.1	208	1Z47	PDB
	potB	37.2	20	Q6MU18	AlphaFold
	potC	119.7	183	4EQB	PDB
Nucleotide uptake	*rnsA*	59.9	146	3DHW	PDB
	rnsB	60.7	81	Q6MUL7	AlphaFold
	rnsC	97.5	176	Q6MUL9	AlphaFold
	rnsD	34.3	5	Q6MUM0	AlphaFold
Amino acid uptake	*oppA*	118.3	315	Q6MU57	AlphaFold
	oppB	46.3	60	Q6MU61	AlphaFold
	oppC	38.0	65	Q6MU60	AlphaFold
	oppD	65.4	242	4FWI	PDB
	oppF	72.3	241	4FWI	PDB
	gltP	57.0	44	6BAT	PDB
	JCVISYN3A_0870	64.3	25	Q6MS63	AlphaFold
	JCVISYN3A_0876	57.7	46	6F2G	PDB
	JCVISYN3A_0878	54.7	8	6LI9	PDB
Export	*JCVISYN3A_0030*	38.8	NA	5LJ9	PDB
	JCVISYN3A_0034	205.6	9	NA	AlphaFold
	JCVISYN3A_0399	200.9	60	NA	AlphaFold
	JCVISYN3A_0401	71.5	65	Q6MTE9	AlphaFold
	JCVISYN3A_0639	159.7	95	NA	AlphaFold
	JCVISYN3A_0691	76.7	12	Q6MSK7	AlphaFold
	ywjA1	77.0	176	2HYD	PDB
	ywjA2	69.8	65	2HYD	PDB
Lipid biosynthesis	*cdsA*	39.8	2	4Q2G	PDB
	clsA	59.9	16	Q6MU83	AlphaFold
	pgpA	31.9	NA	Q6MTZ1	AlphaFold
	pgsA	22.6	37	Q6MS68	AlphaFold
	plsY	28.5	2	5XJ5	PDB
Membrane protein insertion and protein translocation	*JCVISYN3A_0412*	159.1	216	Q6MTD5	AlphaFold
	secA	107.6	574	1M6N	PDB
	secE	12.8	9	Q6MS21	AlphaFold
	secG	10.4	4	NA	AlphaFold
	secY	53.3	66	5EUL	PDB
	ftsH	71.7	148	4EIW	PDB
	ftsY	46.8	89	2XXA	PDB
	lspA	23.6	4	Q6MT29	AlphaFold
	yidC	45.5	120	Q6MRS4	AlphaFold
Unknown	*JCVISYN3A_0005*	42.7	73	Q6MUM3	AlphaFold
	JCVISYN3A_0033	121.6	61	Q6MUJ9	AlphaFold
	JCVISYN3A_0060	31.1	10	Q6MUG6	AlphaFold

	JCVISYN3A_0094	27.4	191	Q6MUE4	AlphaFold
	JCVISYN3A_0116	17.5	63	Q6MUC1	AlphaFold
	JCVISYN3A_0143	57.5	40	Q6MU87	AlphaFold
	JCVISYN3A_0146	28.9	29	Q6MU84	AlphaFold
	JCVISYN3A_0164	19.5	10	Q6MU62	AlphaFold
	JCVISYN3A_0235	11.1	NA	Q6MUJ6	AlphaFold
	JCVISYN3A_0239	71.2	55	Q6MTW6	AlphaFold
	JCVISYN3A_0248	22.7	14	Q6MTV4	AlphaFold
	JCVISYN3A_0249	23.0	53	Q6MTV3	AlphaFold
	JCVISYN3A_0296	25.4	10	Q6MTQ1	AlphaFold
	JCVISYN3A_0317	8.9	20	Q6MTN0	AlphaFold
	JCVISYN3A_0325	58.1	11	Q6MTM0	AlphaFold
	JCVISYN3A_0326	30.6	35	Q6MTL9	AlphaFold
	JCVISYN3A_0332	32.8	32	Q6MTL4	AlphaFold
	JCVISYN3A_0346	27.4	35	Q6MTJ8	AlphaFold
	JCVISYN3A_0353	15.0	69	Q6MTJ1	AlphaFold
	JCVISYN3A_0379	10.8	NA	Q6MTG7	AlphaFold
	JCVISYN3A_0388	24.2	87	Q6MTG0	AlphaFold
	JCVISYN3A_0411	58.1	84	Q6MTD7	AlphaFold
	JCVISYN3A_0478	22.9	9	Q6MT75	AlphaFold
	JCVISYN3A_0516	49.0	63	Q6MT31	AlphaFold
	JCVISYN3A_0538	23.6	408	Q6MT09	AlphaFold
	JCVISYN3A_0592	58.3	79	Q6MSW5	AlphaFold
	JCVISYN3A_0601	36.1	73	Q6MSU7	AlphaFold
	JCVISYN3A_0605	11.5	69	Q6MTM4	AlphaFold
	JCVISYN3A_0696	29.5	4	Q6MSK2	AlphaFold
	JCVISYN3A_0777	18.8	10	Q6MSC0	AlphaFold
	JCVISYN3A_0778	9.7	5	Q6MSA6	AlphaFold
	JCVISYN3A_0797	9.1	9	Q6MS87	AlphaFold
	JCVISYN3A_0827	56.4	212	Q6MS36	AlphaFold
	JCVISYN3A_0830	10.0	8	Q6MS34	AlphaFold
	JCVISYN3A_0852	8.3	NA	Q6MUG9	AlphaFold
	JCVISYN3A_0877	25.7	9	Q6MS70	AlphaFold
	JCVISYN3A_0881	52.8	34	Q6MS76	AlphaFold
	prkC	43.8	1	Q6MTT8	AlphaFold
Other functions	*JCVISYN3A_0338*	25.6	110	Q6MTK6	AlphaFold
	JCVISYN3A_0398	95.4	NA	Q6MTF2	AlphaFold
	JCVISYN3A_0439	80.7	374	Q6MTA9	AlphaFold
	JCVISYN3A_0440	108.4	409	Q6MTA8	AlphaFold
	JCVISYN3A_0481	16.0	9	Q6MT70	AlphaFold
	JCVISYN3A_0505	40.5	252	Q6MT38	AlphaFold
	JCVISYN3A_0622	14.1	NA	Q6MSS6	AlphaFold
	JCVISYN3A_0851	6.7	NA	Q6MTS8	AlphaFold
	rny	57.6	207	Q6MTI6	AlphaFold
	ftsA	45.5	51	Q6MT24	AlphaFold
	JCVISYN3A_0479	65.0	66	Q6MT73	AlphaFold
	JCVISYN3A_0693	36.3	22	Q6MSK5	AlphaFold

METHODS

Encapsulation probability. The number of enzyme molecules per vesicle (*E*) was calculated as a function of the vesicle size. A given internal concentration (ε) was multiplied with the Avogadro constant (N_A, 6.022x10^{23} mol^{-1}) and the internal vesicle volume, as calculated for a sphere of given radius (r).

$$E = \varepsilon \cdot N_A \cdot \frac{4 \cdot \pi \cdot r^3}{3}$$

The cumulative probability (P) of a vesicle to contain one enzyme with a certain (or larger) abundance (x) was determined from the Poisson probability mass function.

$$P = \frac{e^{-\mathrm{E}} \ * \ \mathrm{E}^{x}}{x!}$$

This assumes independent events, a constant event rate over time and no simultaneous event. Further assumptions are that the encapsulation efficiency is 100% and that the internal volume is not affected by the lipid bilayer. The exact probability of a vesicle to contain zero enzymes was determined similarly. The cumulative probability (P_n) of a vesicle to contain n enzymes was obtained by multiplying the independent cumulative probabilities of each enzyme.

$$P_n = P^n$$

Genomic database screening. The functional annotations of genes encoding membrane proteins (113) in JCVI-syn3A[5,6] were retrieved from a dedicated database[1], and the gene products were organized into the metabolic modules: energy conversion (8), physicochemical homeostasis, including ion transport (7), nutrient transport (34), membrane expansion, including lipid synthesis and protein insertion (14), unknown (38), other functions (12). Within each module, gene products encoding different subunits of the same protein complex are clustered together. For example, the ATPase epsilon subunit, annotated as *atpC*, is not a membrane protein but was manually added to this category as it belongs to the F_oF_1-ATPase complex. Similarly, gene products classified as lipoproteins that belong to membrane protein complexes (*thiC*, *potC*, *pstS*, *rnsB*, *oppA*) are included. For the final list of gene products (114), molecular weight, copy number in JCVI-syn3A, and structural information (either the PDB[2] structure of an homolog, or an AlphaFold[3,4] prediction) were retrieved from the database and used for further analysis.

Copy number of full complexes. The oligomeric state of a protein complex is taken into account in calculating the copy number, when it is known or can be inferred from a structure of a homologous protein (the copy number is halved when the protein forms a dimer). When information on the oligomeric state is missing, the protein is assumed to be a monomer.

Planar section area of protein(s) complexes. The planar section area was determined by manually inspecting homolog structures or making use of AlphaFold predictions. The widest distance between two amino acid residues was measured at a 90º angle from the vertical protein axis; for protein complexes with bulkier cytosolic domains, the latter were used in order to avoid steric clashes. The planar section area (*s*) was then calculated by approximating the structure to a cylinder:

$$s(nm^2) = \pi \cdot \left(\frac{D}{2}\right)^2$$

where D is the measured widest distance. The cumulative planar section area (*S*) was calculated as the sum of planar section area of all proteins:

$$S(nm^2) = \sum_{i=0}^{n} s$$

Next, the planar section area of each complex (s_c) was multiplied by the abundance (*c*) of the protein in JCVI-syn3A:

$$s_c(nm^2) = s \cdot c$$

The cumulative planar section area (S_c) was weighted accordingly:

$$S_c(nm^2) = \sum_{i=0}^{n} s_c$$

Relative surface occupancy of protein complexes. A spherical geometry was assumed for calculating the total surface area of vesicles, in agreement with the shape of phospholipid vesicles under iso-osmotic conditions. The total surface area (*A*) was calculated accordingly, for radii (*r*) in the range 0-1000 nm:

$$A(nm^2) = 4 \cdot \pi \cdot r^2$$

The relative surface occupancy of the full complexes was determined as the ratio between the cumulative planar section area and the total surface area:

$$O = \frac{S_c}{A}$$

where O is the relative surface occupancy of all membrane proteins used in the analysis.

Total protein mass. The mass of each gene product was taken from the database[1]. The mass of gene products that are part of oligomeric complexes were scaled accordingly (*e.g.*, the mass of a protein was doubled when it is part of a homodimeric complex); this correction ensures that at least one active complex is counted. The mass of protein complexes for which structural information was not available are assumed to be monomeric. The sum of the mass values (M) represents the total protein mass when one copy is present:

$$M(g) = \sum_{i=0}^{n} m$$

where m is the mass of each gene product. The weighted mass of each gene product was obtained by multiplying with the corresponding copy number:

$$m_c(g) = m \cdot c$$

where m_c is the mass of a gene product times the copy number (c) of the protein, and m is the mass of a gene product; the total protein mass (M_c) is the sum of the individual masses times the copy number:

$$M_c(g) = \sum_{i=0}^{n} m_c$$

Total lipid mass and lipid-to-protein mass ratio. The surface area available to lipids was determined by subtracting the protein surface occupied by protein complexes (see section above) from the surface areas of spheres with radii in the range 0-1000 nm; the bilayer arrangement was taken into account by including both inner and outer leaflets:

$$A_{out}(nm^2) = (4 \cdot \pi \cdot r^2)(1 - O)$$

$$A_{in}(nm^2) = (4 \cdot \pi \cdot (r - 4)^2)(1 - O)$$

where A_{out} and A_{in} are the outer and inner leaflet surface areas, respectively, r is the radius in the range 0-1000 nm and O is the relative surface occupancy of proteins (see above); a correction factor was applied to the inner radius to account for the approximate bilayer thickness of di-oleoyl phospholipids (~4 nm)[7]. The total lipid surface area (B) was obtained from the sum of outer and inner leaflet surface areas:

$$B(nm^2) = A_{out} + A_{in}$$

Given a 25:25:50 mol:mol:mol DOPE:DOPG:DOPC composition of the vesicles, the total number of phospholipids was determined and converted into mass:

$$L(g) = \frac{B \cdot w}{p \cdot N_A}$$

where L is the total lipid mass, p is the weighted phospholipid surface area (0.63 nm^2, calculated from DOPE[8]=0.524 nm^2, DOPC[8]=0.651 nm^2, DOPG[9]=0.694 nm^2), w is the weighted molecular weight (777.8 g/mol) and N_A is the Avogadro's constant (6.022×10^{23} mol^{-1}). The total lipid mass obtained for each radius was divided by the total protein mass values (see above) to yield the lipid-to-protein mass ratio:

$$LPR = \frac{L}{M_c}$$

SUPPLEMENTARY REFERENCES

(1) Pedreira, T.; Elfmann, C.; Singh, N.; Stülke, J. SynWiki: Functional Annotation of the First Artificial Organism Mycoplasma Mycoides JCVI-Syn3A. *Protein Science* **2022**, *31* (1), 54–62. https://doi.org/10.1002/PRO.4179.

(2) Berman, H. M.; Westbrook, J.; Feng, Z.; Gilliland, G.; Bhat, T. N.; Weissig, H.; Shindyalov, I. N.; Bourne, P. E. The Protein Data Bank. *Nucleic Acids Res* **2000**, *28* (1), 235–242. https://doi.org/10.1093/NAR/28.1.235.

(3) Jumper, J.; Evans, R.; Pritzel, A.; Green, T.; Figurnov, M.; Ronneberger, O.; Tunyasuvunakool, K.; Bates, R.; Žídek, A.; Potapenko, A.; Bridgland, A.; Meyer, C.; Kohl, S. A. A.; Ballard, A. J.; Cowie, A.; Romera-Paredes, B.; Nikolov, S.; Jain, R.; Adler, J.; Back, T.; Petersen, S.; Reiman, D.; Clancy, E.; Zielinski, M.; Steinegger, M.; Pacholska, M.; Berghammer, T.; Bodenstein, S.; Silver, D.; Vinyals, O.; Senior, A. W.; Kavukcuoglu, K.; Kohli, P.; Hassabis, D. Highly Accurate Protein Structure Prediction with AlphaFold. *Nature 2021* **2021**, *596* (7873), 583–589. https://doi.org/10.1038/s41586-021-03819-2.

(4) Varadi, M.; Anyango, S.; Deshpande, M.; Nair, S.; Natassia, C.; Yordanova, G.; Yuan, D.; Stroe, O.; Wood, G.; Laydon, A.; Zídek, A.; Green, T.; Tunyasuvunakool, K.; Petersen, S.; Jumper, J.; Clancy, E.; Green, R.; Vora, A.; Lutfi, M.; Figurnov, M.; Cowie, A.; Hobbs, N.; Kohli, P.; Kleywegt, G.; Birney, E.; Hassabis, D.; Velankar, S. AlphaFold Protein Structure Database: Massively Expanding the Structural Coverage of Protein-Sequence Space with High-Accuracy Models. *Nucleic Acids Res* **2022**, *50* (D1), D439–D444. https://doi.org/10.1093/NAR/GKAB1061.

(5) Hutchison, C. A.; Chuang, R. Y.; Noskov, V. N.; Assad-Garcia, N.; Deerinck, T. J.; Ellisman, M. H.; Gill, J.; Kannan, K.; Karas, B. J.; Ma, L.; Pelletier, J. F.; Qi, Z. Q.; Richter, R. A.; Strychalski, E. A.; Sun, L.; Suzuki, Y.; Tsvetanova, B.; Wise, K. S.; Smith, H. O.; Glass, J. I.; Merryman, C.; Gibson, D. G.; Venter, J. C. Design and Synthesis of a Minimal Bacterial Genome. *Science (1979)* **2016**, *351* (6280), aad6253. https://doi.org/10.1126/SCIENCE.AAD6253.

(6) Breuer, M.; Earnest, T. M.; Merryman, C.; Wise, K. S.; Sun, L.; Lynott, M. R.; Hutchison, C. A.; Smith, H. O.; Lapek, J. D.; Gonzalez, D. J.; Crécy-Lagard, V. de; Haas, D.; Hanson, A. D.; Labhsetwar, P.; Glass, J. I.; Luthey-Schulten, Z. Essential Metabolism for a Minimal Cell. *Elife* **2019**, *8*, e36842. https://doi.org/10.7554/ELIFE.36842.

(7) Rawicz, W.; Olbrich, K. C.; Mcintosh, T.; Needham, D.; Evans, E. Effect of Chain Length and Unsaturation on Elasticity of Lipid Bilayers. *Biophys J* **2000**, *79* (1), 328–339.

(8) Orsi, M.; Essex, J. W. Physical Properties of Mixed Bilayers Containing Lamellar and Nonlamellar Lipids: Insights from Coarse-Grain Molecular Dynamics Simulations. *Faraday Discuss* **2013**, *161*, 91–111. https://doi.org/10.1039/c2fd20110k.

(9) Pan, J.; Heberle, F. A.; Tristram-Nagle, S.; Szymanski, M.; Koepfinger, M.; Katsaras, J.; Kučerka, N. Molecular Structures of Fluid Phase Phosphatidylglycerol Bilayers as Determined by Small Angle Neutron and X-Ray Scattering. *iochimica et Biophysica Acta (BBA)-Biomembranes* **2012**, *1818* (9), 2135–2148. https://doi.org/10.1016/J.BBAMEM.2012.05.007.
